# SCARN a Novel Class of SCAR Protein That Is Required for Root-Hair Infection during Legume Nodulation

**DOI:** 10.1371/journal.pgen.1005623

**Published:** 2015-10-30

**Authors:** Liping Qiu, Jie-shun Lin, Ji Xu, Shusei Sato, Martin Parniske, Trevor L. Wang, J. Allan Downie, Fang Xie

**Affiliations:** 1 National Key Laboratory of Plant Molecular Genetics, Institute of Plant Physiology and Ecology, Shanghai Institutes for Biological Sciences, Chinese Academy of Sciences, Shanghai, China; 2 Kazusa DNA Research Institute, Kisarazu, Japan; 3 Graduate School of Life Sciences, Tohoku University, Sendai, Japan; 4 University of Munich LMU, Faculty of Biology, Martinsried, Germany; 5 John Innes Centre, Norwich, United Kingdom; University of Aarhus, DENMARK

## Abstract

Rhizobial infection of legume root hairs requires a rearrangement of the actin cytoskeleton to enable the establishment of plant-made infection structures called infection threads. In the SCAR/WAVE (Suppressor of cAMP receptor defect/WASP family verpolin homologous protein) actin regulatory complex, the conserved N-terminal domains of SCAR proteins interact with other components of the SCAR/WAVE complex. The conserved C-terminal domains of SCAR proteins bind to and activate the actin-related protein 2/3 (ARP2/3) complex, which can bind to actin filaments catalyzing new actin filament formation by nucleating actin branching. We have identified, *SCARN* (*SCAR-Nodulation*), a gene required for root hair infection of *Lotus japonicus* by *Mesorhizobium loti*. Although the SCARN protein is related to *Arabidopsis thaliana* SCAR2 and SCAR4, it belongs to a distinct legume-sub clade. We identified other SCARN-like proteins in legumes and phylogeny analyses suggested that SCARN may have arisen from a gene duplication and acquired specialized functions in root nodule symbiosis. Mutation of *SCARN* reduced formation of infection-threads and their extension into the root cortex and slightly reduced root-hair length. Surprisingly two of the *scarn* mutants showed constitutive branching of root hairs in uninoculated plants. However we observed no effect of *scarn* mutations on trichome development or on the early actin cytoskeletal accumulation that is normally seen in root hair tips shortly after *M*. *loti* inoculation, distinguishing them from other symbiosis mutations affecting actin nucleation. The C-terminal domain of SCARN binds to ARPC3 and ectopic expression of the N-terminal SCAR-homology domain (but not the full length protein) inhibited nodulation. In addition, we found that *SCARN* expression is enhanced by *M*. *loti* in epidermal cells and that this is directly regulated by the NODULE INCEPTION (NIN) transcription factor.

## Author Summary

Characterization of *Lotus japonicus* mutants defective for nodule infection by rhizobia led to the identification of a gene we named *SCARN*. Two of the five alleles caused formation of branched root-hairs in uninoculated seedlings, suggesting SCARN plays a role in the microtubule and actin-regulated polar growth of root hairs. SCARN is one of three *L*. *japonicus* proteins containing the conserved N and C terminal domains predicted to be required for rearrangement of the actin cytoskeleton. *SCARN* expression is induced in response to rhizobial nodulation factors by the NIN (NODULE INCEPTION) transcription factor and appears to be adapted to promoting rhizobial infection, possibly arising from a gene duplication event. SCARN binds to ARPC3, one of the predicted components in the actin-related protein complex involved in the activation of actin nucleation.

## Introduction

In most eukaryotic cells, the reversible association of G-actin subunits can result in the dynamic formation of actin filaments that play essential roles in changing cell shape and controlling nuclear localization. In addition, actin filaments form a framework along which vesicles and proteins can be trafficked to localized sites on the plasma membrane. In non-plant cells, cellular protrusions of the plasma membrane are critical for cell migration and are induced by polymerization and branching of actin [1]. A complex containing actin-related proteins 2/3 (ARP2/3) induces nucleation of branched actin that can drive the cellular protrusions. In the plant kingdom, the actin nucleation activity of the ARP2/3 complex is solely regulated by the SCAR/WAVE (Suppressor of cAMP receptor defect/WASP family verprolin-homologous protein) complex [1].

Due to the presence of the cell wall, membrane protrusions do not normally occur in plant cells, but nevertheless there are highly dynamic changes to the actin filaments catalyzed by nucleation of actin branching and actin polymerization and de-polymerization [2]. However, in contrast to non-plant cells, cytoskeletal control of plant-cell shape must be indirect, primarily mediated by the delivery of cell-wall remodeling proteins to localized sites in the cell periphery [3]. The SCAR/WAVE-ARP2/3 system plays an important role in polar growth of some plant cells such as trichomes and root hairs where mutations cause developmental phenotypes.

In legumes, identification of infection-defective mutants has revealed that there is a special requirement for the SCAR/WAVE-ARP2/3 system during nodule infection. During the bacterial initiation of nitrogen-fixing nodules on the roots of legumes, the rhizobia gain entry to the root via plant-made structures called infection threads along which the bacteria grow [4]. The infection thread, which is usually initiated in a root hair, is essentially an intracellular tube formed by an invagination of the plant cell wall and membrane [4,5]. This requires a new type of inwardly-directed polar growth that appears to be controlled by the position of the root-hair nucleus [6]. Based on the patterns of induction of cell-cycle related genes and analysis of nuclear enlargement, infection thread growth appears to be associated with the activation of a partial cell cycle that involves nuclear endoreduplication but not nuclear division [7].

Establishment of infection threads in root hairs requires rhizobial production of signals called Nod factors. The first plant morphological response to Nod factors is for the root hair to deform, sometimes curling back on itself, enclosing the bacteria in an infection pocket; this involves rearrangements in the actin cytoskeleton [8–11]. A transient increase in rapidly-growing actin microfilaments is induced in root hairs 2–5 min after Nod-factor addition [12]. After rhizobial entrapment, a new phase of development in the root hair leads to the initiation of growth of the infection thread [13,14] and this involves a second phase of redistribution of actin polymerization sites, this time to the point of rhizobial entry [12]. As the infection proceeds, a second developmental program is activated in the root cortex leading to morphogenesis of the nodule structure, that will eventually be infected following the extended growth of the infection threads through the root hair cells into the root cortex [15,16].

Critical to the activation of the infection and nodule development programs is a signaling pathway activated by rhizobial Nod factors [17,18]. Following Nod-factor binding to plasma-membrane receptors, calcium oscillations are induced in and around the root-hair nuclei and these oscillations are detected by a calcium and calmodulin-dependent kinase that activates a transcription factor called CYCLOPS [19,20]. Downstream of CYCLOPS there is activation of another transcription factor NIN, which is needed for infection-pocket and infection-thread development [21]. After rhizobial entrapment, a second signaling pathway is activated; this appears to require both higher levels and higher structural specificity of Nod factors and induces a calcium influx across the plasma membrane; if the appropriate host-specific decorations are absent from the Nod factors, both the calcium influx and infection thread initiation are significantly reduced [22,23]. It has been suggested that this pathway may be activated by the ROP-GAP pathway and is also related to the production of reactive oxygen species via an NADPH oxidase on the plasma membrane [18,22]. Although there is high signaling specificity, it is evident that some endophytic rhizobia-like bacteria (lacking *nod* genes) can gain entry using infection threads initiated by nodulation-competent rhizobia[24].

Several nodulation-defective mutants have been identified in which infection pockets are initiated or established, but most infection thread growth is blocked; some of these mutations are in regulatory genes such as, *NIN*, *LIN*, *RPG*, *ERN1* and *NFYA1* [21,25–29]. However other mutations are in genes that appear likely to be involved with structural requirements associated with infection thread initiation. One of these is in a nodulation-specific pectate lyase gene *(NPL)* the product of which is thought to locally degrade plant cell walls to allow initiation of infection thread growth. The *NPL* gene is directly regulated by NIN in response to Nod factor signaling [30]. Three mutants with similar phenotypes to the *npl* mutant are in the genes *NAP* (for Nck-associated protein 1) [31,32], *PIR* (for 121F-specific p53 inducible RNA) [31] and *ARPC1* (for actin related protein complex 1) [33]. These proteins participate in promoting actin nucleation via the SCAR/WAVE-ARP2/3 complex. One possibility is that the SCAR/WAVE-ARP2/3 complex directs to the sites of infection thread initiation and growth, the cell-wall degrading enzymes (such as NPL) and the cell-wall and cell-membrane synthesis enzymes that will be required to establish an infection thread. If this model is correct, analysis of genes required for infection-thread initiation may give us insights not only into how legumes accommodate infection by rhizobia, but also into aspects of how the SCAR/WAVE-ARP2/3 complex operates and can be adapted for special situations in plant development.

One of the questions with regard to the operation of the SCAR/WAVE-ARP2/3 complex in legume infection is whether it simply uses existing components or whether there is induction of components to facilitate rhizobial infection. In this work, we have identified a novel gene *SCARN* (*SCAR-Nodulation*) required for initiation of infection threads and this gene contains domains typical of SCAR proteins. However, although the gene product is conserved in legumes, it is novel because it is not closely related to any of the SCAR-family proteins identified in Arabidopsis. This gene is directly regulated by the nodulation transcription factor NIN. Intriguingly, two of the mutations in *SCARN* resulted in constitutive induction of root-hair branching, a phenotype normally induced by Nod-factors.

## Results

### Identification of four alleles of a gene required for legume infection by rhizobia

To understand the molecular mechanisms of infection thread formation during rhizobial infection of *L*. *japonicus* roots, we screened an ethyl-methane-sulfonate (EMS) mutagenized population [34–36] of *L*. *japonicus* Gifu B-129 for defects in infection by *M*. *loti*. Mutants defective for nitrogen fixation were initially identified based on nitrogen starvation (yellowing) of the leaves of plants grown under limiting nitrogen and then screening for the absence of nodules, or the presence of white nodules, indicative of an ineffective symbiosis. Candidate mutants were then inoculated with a strain of *M*. *loti* expressing a β-galactosidase (*lacZ*) gene and nodules formed were then stained with X-gal to determine if they were, or were not infected. The various mutants that appeared to have mostly uninfected nodules were crossed with the wild-type Miyakojima (MG20) to generate mapping populations. Rough mapping using established DNA markers http://www.kazusa.or.jp/lotus/ revealed that four of the mutations were localized on *L*. *japonicus* linkage group 3, between markers TM0111 and TM0707. These mutations were in lines SL2654-3, SL5737-2, SL6119-2 and SL1058-2 and the F_2_ populations generated from crosses with MG20 were scored for nodulation defects. All of the mutations segregated 3:1 (p < 0.05) based on the numbers (shown in parentheses) of WT and mutant plants in the F_2_ populations from crosses with SL2654-3 (246 Nod^+^/78 Nod^-^, x^2^ value = 0.0337); SL5737-2 (144 Nod^+^/33 Nod^-^, x^2^ value = 1.8165); SL6119-2 (236 Nod^+^/67 Nod^-^, x^2^ value = 0.5631) and SL1058-2 (200 Nod^+^/59 Nod^-^, x^2^ value = 0.244). These data suggested that we had identified four allelic monogenic recessive mutations and so allelism was tested in reciprocal crosses. When SL2654-3 was crossed with SL5737-2, SL1058-2 and SL6119-2, or SL6119-2 was crossed with SL5737-2, all the F_1_ plants produced no nodules or only small white bumps (S1 Table). The results of these reciprocal crosses confirmed that the four mutations are allelic, and so fine mapping for positional cloning and analyses of phenotypes were done primarily with one mutant (SL2654-3).

### Identification of a mutation in a gene encoding a novel SCAR homolog

**Fig 1:**
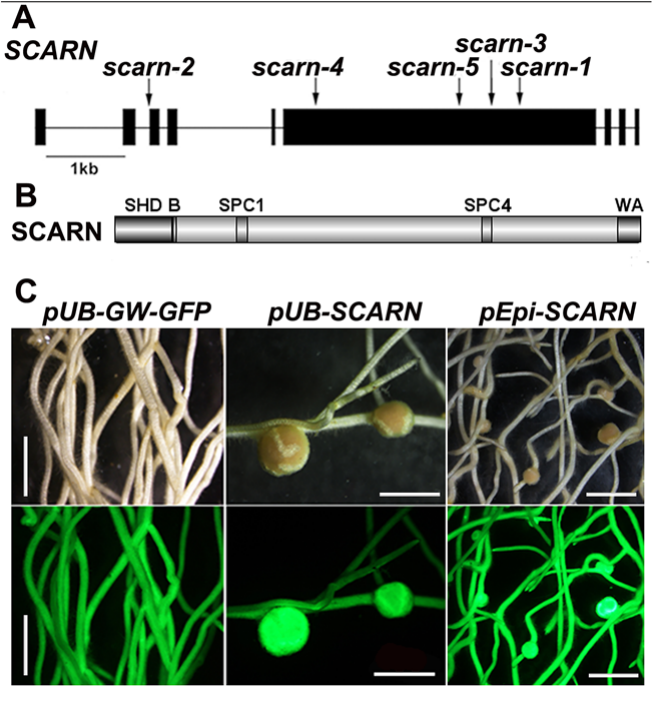
*SCARN* gene and protein structure and complementation of *scarn-1*. **(A)** The *SCARN* gene exons are shown as black boxes. The locations of the mutations in SL2564-3 (*scarn-1)*, SL5737-2 (*scarn-2)*, SL6119-2 (*scarn-3)*, SL1058-2 (*scarn-4)* and the LORE mutant *(scarn-5)* are shown. **(B)** The SCARN protein has an N-terminal SCAR homology domain (SHD) and C-terminal WH2 and Acidic (WA) domains. Between the SHD and WA domains there are two plant-specific conserved SCAR motifs SPC1 and SPC4. **(C)** Hairy roots of the *scarn-1* mutant transformed by *A*. *rhizogenes* with the control plasmid (*pUb-GW-GFP*), and complementation of nodulation by the same plasmid containing the *SCARN* genomic DNA downstream of the ubiquitin (*pUb*:*SCARN*), or of the epidermal-specific promoter (*pEpi*:*SCARN*). The upper panels show bright field images and the lower panels are epifluorescence microscopy images showing GFP expression in the same transgenic roots. Scale bar 2mm.

From crosses between SL2654-3 and MG20, 439 mutant progeny were identified. Genotyping of these mutants delimited the mutated locus in SL2654-3 between markers TM1465 and TM0116; there was no recombination with marker BM2233 (S1 Fig). We analyzed the predicted protein sequences of genes in this region and identified a large gene composed of nine exons (Fig 1A) encoding a predicted 1627 amino-acid protein, that has domains with similarity to domains typically found in SCAR (suppressor of cAMP receptor) proteins. SCAR proteins are components of the SCAR/WAVE complex, which is known to participate in the regulation of actin nucleation in *Arabidopsis thaliana* [37]. Since other components of the SCAR/WAVE complex had previously been identified as being required in legumes for root-hair infection by rhizobia, we thought the gene encoding a protein with domains showing similarity to SCAR protein was a good candidate. Amplification and sequencing of this gene from SL2654-3 revealed a C-T transition, leading to a stop codon replacing Q1199. We amplified and sequenced the gene from the other three mutants and all had mutations (Table 1 and Fig 1A): SL5737-2 has a G-A transition at the third exon splice acceptor site, SL6119-2 has a C- T transition causing a premature stop replacing at Q1100, and SL1058-2 has 1 base pair deletion in the sixth exon causing a frame shift. Some time after the detailed analyses of these mutants, we also obtained a LORE1 insertion (30053577) in this gene from the *L*. *japonicus* LORE1 retrotransposon mutagenesis pool at Aarhus University, Denmark [38,39]; this mutant was also defective for infection and nodulation.

**Table 1:** *L*. *japonicus scarn* mutant alleles.

Allele (Previous name)	Mutation	Reading Frame Change	
*scarn-1* (SL2654-3)	C_6148_→T		Q_1199_→stop
*scarn-2* (SL5737-2)	G_1476_→A	Splice site mutation	
*scarn-3* (SL6119-2)	C_5851_→T	Q_1100_→stop	
*scarn-4* (SL1058-2)	1bp deletion in exon 6 (position 3580)	Frame shift	
*scarn-5* (30053577)	LORE-1 insertion in exon 6 (position 5421)	Insertion of 20 amino acids after R956→ premature stop	

The wild-type gene was amplified from the genome of Gifu B-129 and cloned behind the ubiquitin promoter in the vector pUB-GW-GFP [40]. The construct was introduced into roots of *SL2654-3* by *Agrobacterium rhizogenes*-mediated hairy root transformation. Normal nodulation was restored in the *SL2654-3* mutant (Fig 1C and Table 2). Taken together, it is clear from the multiple alleles and complementation data, that mutations in the *SCAR-*like gene caused the defect in nodule infection. As described below, although there are conserved domains, the overall predicted protein sequence was not strongly homologous to any of the Arabidopsis SCAR proteins. Furthermore the identified gene is increased in expression during nodule infection and so we named the gene *SCARN* (*SCAR-Nodulation*). Accordingly we named the alleles in SL2654-3, SL5737-2, SL6119-2 and SL1058-2 *scarn-1*, *scarn-2*, *scarn-3* and *scarn-4* respectively and we named the *LORE1* retrotransposon mutant allele *scarn-5* (Table 1).

**Table 2:** Complementation of *scarn-1* nodulation phenotype by hairy root transformation.

Line	Transformation construct	Nodulation Ratio^a^	Nodules (SD)^b^
Wild type	Empty vector	14/15	3.5 (2.6)
*scarn-1*	Empty vector	0/17	0
Wild type	*pUb-SCARN*	16/18	3.2 (2.5)
*scarn-1*	*pUb-SCARN*	12/18	3.5 (3.3)
*scarn-1*	*pEpi-SCARN*	11/11	6.5 (5.4)

^a^ Ratio indicates numbers of successfully transformed plants that formed nodules versus all plants of the indicated line that were successfully transformed with the indicated construct.

^b^ Mean nodule number per successfully transformed plant.

### *scarn* mutants produce uninfected nodules but are infected by a mycorrhizal fungus

**Fig 2:**
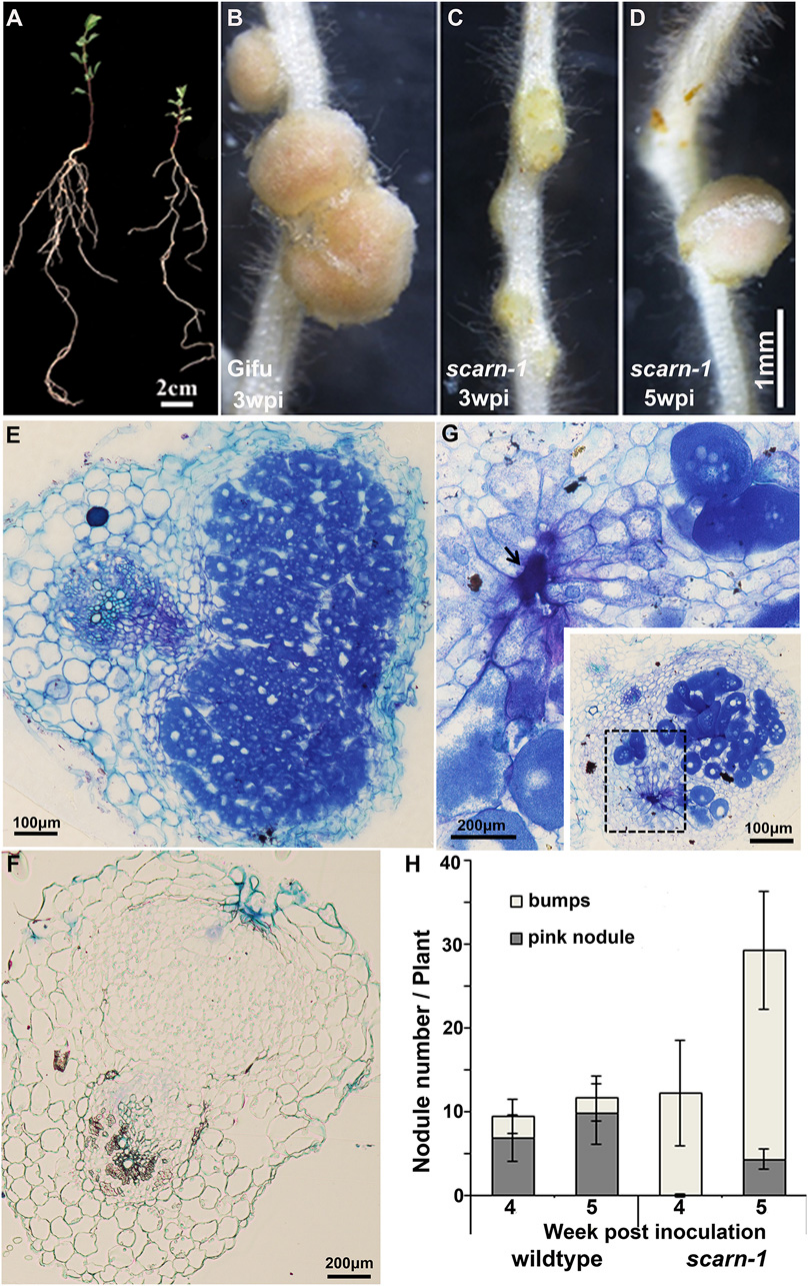
Phenotype of the *L*. *japonicus scarn-1* mutant. **(A)** Whole plants of wild-type Gifu B-129 and *scarn-1* five weeks after germination and growth in a 1:1 mix of perlite and vermiculite inoculated with *M*. *loti* R7A. Left: wild-type Gifu B-129; right: SL2564-3 (*scarn-1)*. **(B-C)** Nodules formed 3 weeks after *M*. *loti* inoculation of (B) wild type or (C) SL2564-3 (*scarn-1)*. **(D)** A nodule formed on *scarn-1* 5 weeks after inoculation. Panels E, F and G show light microscopy sections of the nodules shown in B, C and D respectively. Arrow in (G) shows the pockets of intercellular bacteria. **(H)** Nodule numbers 4 and 5 weeks after inoculation of wildtype and SL2564-3 *(scarn-1)* plants inoculated with *M*. *loti* R7A (the bar indicated 95% confidence intervals): grey columns: mature, pink nodules; white columns: white immature nodule bumps. Scale bars: A, 2 cm; B-D, 1 mm; and E, 100 μm; F-G, 200μm.

Although the *scarn* mutants showed symptoms of nitrogen-starvation on nitrogen-deficient nutrient medium (Fig 2A), they grew normally when supplied with nitrate (S2A Fig). All five *scarn* mutants produced small white nodules (hereafter called bumps) three weeks after inoculation with *M*. *loti* (Fig 2C and S2B Fig). The *scarn-5* mutant had the most severe phenotype, producing only a couple of white nodule-like bumps four weeks after inoculation (S2C Fig). However, 4–5 weeks after inoculation, some *scarn* mutants produced a few pale-pink nodules (Fig 2D). Sections of the bumps and pale-pink nodules were examined by light microscopy showing that the bumps were uninfected nodules (Fig 2F), whereas the wild type formed fully infected nodules (Fig 2E). The occasional pale-pink nodules were partially infected and some had the characteristic ‘butterfly’ structure (Fig 2G), indicative of infections via intercellular entry [41]. The net effects of the *scarn* mutations on nodulation were to greatly reduce the numbers of pink nodules and to induce the formation of more white bumps compared with wild type plants (Fig 2H). Since the *scarn1-4* mutants were similar, and we did not obtain the *scarn-5* mutant until relatively late in the project, we used the *scarn-1* mutant in all subsequent experiments except where noted.

Some legume nodulation mutants are also defective for the arbuscular mycorrhizal symbiosis. Therefore, we also scored the *scarn-1* mutant for infection by the mycorrhizal fungus *Glomus intraradices*. Normal infection and arbuscule formation was observed (S3A–S3C Fig).

### Mutation of *scarn* blocked infection-thread growth but not induction of early nodulation genes

**Fig 3:**
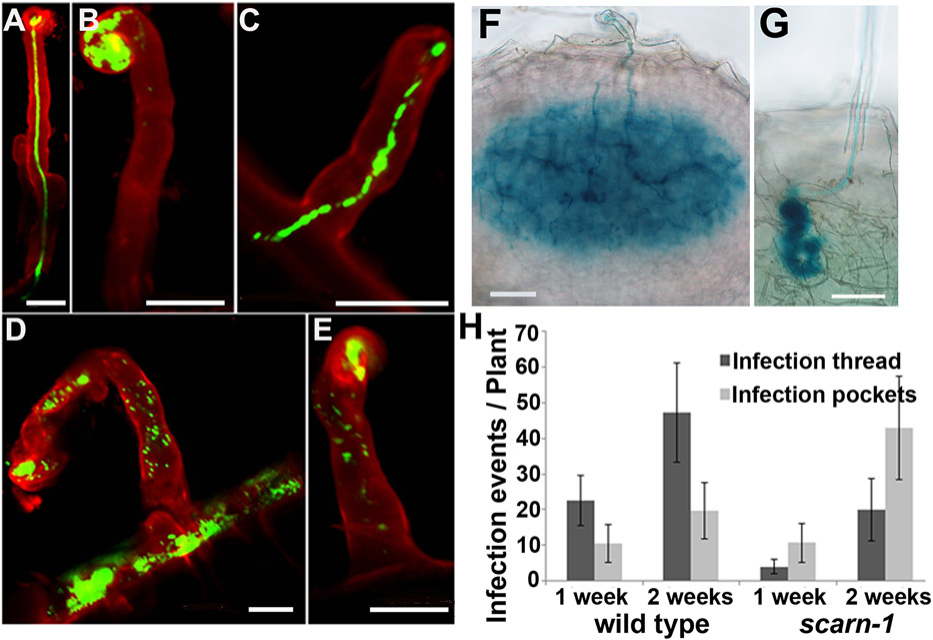
Infection thread phenotype of the *scarn-1* mutant (SL2564-3). **(A-E)** Confocal microscopy of root hairs in wild type (A) or *scarn-1* (B-E) 1 week after inoculation with *M*. *loti* R7A expressing GFP. The green fluorescence shows a normal infection thread in a curled root hair (A), whereas most of infection events were blocked in infection foci of the *scarn-1* mutant (B). Sometimes, rhizobia were observed within root hairs but outside of infection threads (D, E). **(F-G)** Histochemical staining (X-gal) of an infection 1 week after inoculation of wild type (F) or one of the rare infection threads in SL2564-3 (*scarn-1)* that penetrated beyond the root-hair cell (G). Scale bars: A-E, 20 μm; F-G, 50 μm. **(H)** Histogram showing the average numbers (with 95% confidence levels) of infection threads plant in the wild type and the *scarn-1* mutant 1 and 2 weeks after inoculation with *M*. *loti* R7A/*lacZ*. Infections were counted microscopically using roots stained with X-gal.

The absence of bacteria in most of the nodules of the *scarn* mutants suggested that rhizobial infection was abnormal. Assays of infection by *M*. *loti* constitutively expressing a green fluorescent protein (GFP) or a β-galactosidase (*lacZ*) marker gene showed that the *scarn-1* mutant was defective for infection thread formation. Unlike the wild-type, in which infection threads initiated from curled root hairs and extended through the epidermal cells (Fig 3A), most of the infections of *scarn-1* were blocked at the stage of formation of infection pockets (Fig 3B) and occasionally, some infection threads grew within root hairs of the *scarn-1* mutant (Fig 3C). In some of the few infections that did occur in the mutant, it appeared that the bacteria were released into the root hair cell (Fig 3D and 3E), a phenomenon that has been noted in several infection mutants [42]. After 5–7 days, infection threads in wild-type plants ramified into the cortex (Fig 3F), but in the *scarn-1* mutant, although occasional infection threads extended to the base of the root hair, they did not extend deep into the cortex (Fig 3G). Analyses of infection of the *scarn-1* mutant 1 and 2 weeks after inoculation (Fig 3H) revealed that the total number of initiated infections was not reduced in the *scarn-1* mutant, but most of these events were arrested in the infection foci in root hairs.

During the initiation of the rhizobial-legume symbiosis, several genes including *NIN*, *NPL* and *ENOD40-1*, are induced in response to rhizobia or Nod factors. These genes may be involved in rhizobial infection and/or nodule organogenesis and so we used quantitative real-time PCR to assay their induction in the *scarn-1* mutant by *M*. *loti*. Five days after inoculation with *M*. *loti*, the *NIN*, *ENOD40-1* and *NPL* genes were strongly and similarly induced in both the *scarn-1* mutant and wild-type (S4 Fig) showing that *scarn* is not required for their expression.

### Mutations in *scarn* affect root-hair development and *M*. *loti*-induced root hair deformation but not trichome formation

In *Arabidopsis thaliana*, mutations in *SCAR2* cause distorted trichomes (*AtSCAR2* is also called *DISTORTED3* or *IRREGULAR TRICHOME BRANCH1*) [43,44]. Mutations in some other components (*nap*, *pir* and *arpc1*) of the SCAR/WAVE-ARP2/3 complex in legumes also cause distorted trichomes, and affect root-hair growth and pod and seed development [31–33]. Visual inspection did not reveal obvious differences in trichomes in the *scarn* mutants (S5A Fig) and scanning electron microscopy revealed that the trichomes in wild type and *scarn-1* were indistinguishable (S5B Fig). Moreover, there were no apparent defects in pod or seed development of *scarn* mutants compared with wild type (S5C Fig).

**Fig 4:**
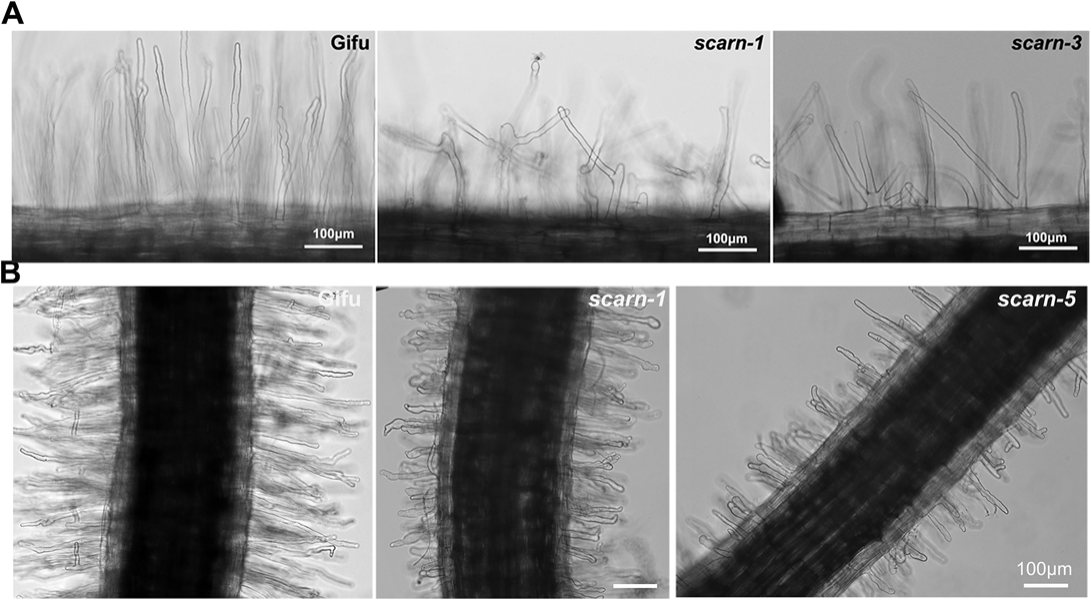
Root hair and *M*. *loti*-induced root hair deformation in *L*. *japonicus* wildtype (Gifu) and *scarn* mutants (*scarn-1* and *scarn-5*). **(A)** Light microscopy image of the branched root hairs in the mature zone of wild type (left) and *scarn-1* (middle) and *scarn-3* (right) following 2 days of growth on uninoculated FP liquid medium. The root hair length of mature zone of wildtype and *scarn* mutants are similar, (measured about 1.0–1.5 cm to the root hair tip). Scale bar, 100 μm. **(B)** Light microscopy images of *M*. *loti* R7A-induced root-hair deformation in the infection zone of wild type (Gifu), *scarn-1* and *scarn-5* mutants. Scale bar, 100 μm

The *scarn* mutants formed slightly shorter root hairs than wild type (S5D Fig). Interestingly, the *scarn-1* and *scarn-3* mutants produced branched root hairs in the mature zone even in the absence of *M*. *loti* (Fig 4A).

We assayed *M*. *loti*-induced root-hair deformation with the *scarn* mutants and saw that they had altered root hair deformation, with more branching and swelling of the root hairs (Fig 4B). It appeared that the root hair swelling was associated with reduced root hair growth. These observations suggest that *SCARN* is required for both normal tip growth following exposure to *M*. *loti* and for the establishment of polar growth of infection threads. The other subunits of the SCAR/WAVE complex, LjPIR, LjNAP, MtNAP and LjARPC1, are not only required for rhizobial infection, but are also required for normal trichome and root-hair growth, and seed development, as observed with their homologues in Arabidopsis. However, unlike the *NAP*, *PIR* and *ARPC1* genes, *SCARN* is not required for the development of trichomes or normal pods and seeds in legumes.

### Expression pattern of *SCARN*

**Fig 5:**
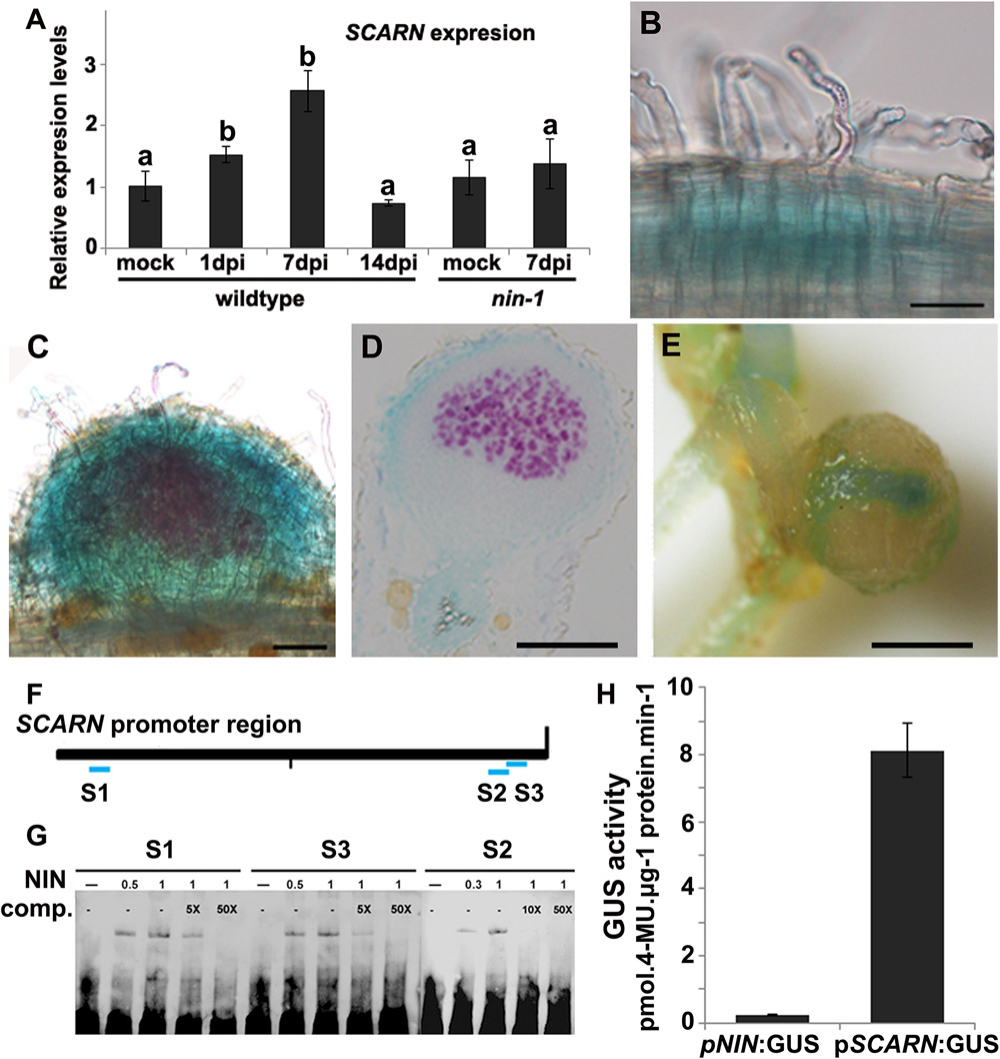
*SCARN* expression in *L*. *japonicus* roots, and NIN directly induces *SCARN* expression. **(A)** Quantitative RT-PCR analysis of *SCARN* expression in mock inoculated (mock) and *M*. *loti* inoculated roots of wild-type (1, 7, 14 days) and *nin-1* mutant (7 days) roots of *L*. *japonicus* after inoculation of seedlings growth in perlite/vermiculite. Different letters indicate significant differences (Student-Newman-Kuels test, P < 0.05). **(B-E)** Histochemical localization of GUS activity in *A*. *rhizogenes*-induced hairy roots transformed with p*SCARN*:GUS. The hairy roots were analyzed 7 days (B-D) and 14 days (E) after inoculation with *M*. *loti* R7A/*lacZ* and stained with both Magenta-Gal and X-Gluc to visualize *M*. *loti* (in purple) and *pSCARN*:GUS expression (in blue). (B) Shows *pSCARN*:GUS expression in the root below *M*. *loti*-infected root hairs. (C) Shows *pSCARN*:GUS expression in a whole stained young nodule, and *M*. *loti* in an infection thread and young nodule (purple). (D) Shows *pSCARN*:GUS expression in the epidermal cells of a section of a nodule in which some the nodule cells are infected by *M*. *loti* as seen by the magenta staining. (E) Shows *pSCARN*:GUS expression limited mostly to the vascular bundles of a mature nodule. (B-E): Bar = 100 μm. **(F)** Map of the *SCARN* promoter region showing regions S1, S2 and S3 that show sequence homology to NIN binding sites. This entire region was used to make the *pSCARN*-promoter GUS fusion. **(G)** The S1, S2 and S3 regions marked in blue in the predicted SCARN promoter (F) were used as probes for three electrophoresis-mobility-shift assays. In each there was 0.5, 1 μg or no NIN protein added as indicated. In two lanes of each assay, a 5x, 10x or 50x excess (as indicated) of unlabeled competitor DNA fragment was added. **(H)** Transactivation assay in *N*. *benthamiana* leaves showing that NIN can activate *pSCARN*:GUS expression. The *pNIN*:GUS fusion was used as a negative control. GUS activity was determined histochemically and quantitatively in leaf discs. Mean values and standard deviations were determined from three biological replicates.

We analyzed *SCARN* transcript levels in different organs by quantitative real-time PCR and found no significant difference in expression in shoots, leaves, flowers and roots (S6A Fig). We analyzed *SCARN* expression in roots at different time points after inoculation with *M*. *loti*: *SCARN* expression was slightly increased 1 and 7 days after inoculation, but its expression returned to basal levels 14 days after inoculation (Fig 5A). This suggested that *SCARN* expression is enhanced during rhizobial infection. To further investigate this we analyzed the spatial and temporal expression of *SCARN* by generating *A*. *rhizogenes-*induced transgenic hairy roots carrying the β-glucuronidase (GUS) gene behind the *SCARN* promoter (*pSCARN*:*GUS)*. When inoculated with *M*. *loti*, we detected weak expression in epidermal cells under infection foci or elongated infection threads in the transformed roots (Fig 5B) confirming that *SCARN* is up-regulated during rhizobial infection. The strongest GUS expression was detected in young nodules (Fig 5C and S6B Fig), sections of which indicated there was GUS activity in the epidermal and outer cortical cells but not in the central tissue (Fig 5D). The observation that the *scarn-1* and *scarn-3* alleles caused root-hair branching (Fig 4A) implies that SCARN must be expressed in root hairs. Furthermore, the altered pattern of *M*. *loti*-induced root-hair deformation also implies that SCARN must be expressed in root hairs. However the *SCARN* expression must be below the level of detection by the *pSCARN*:GUS fusion. In mature nodules (about 2 week post inoculation), the GUS activity was primarily seen at nodule vascular bundles (Fig 5E and S6B Fig). In the absence of *M*. *loti*, *pSCARN*:*GUS* expression was detected in primary and lateral root tips, and sites of lateral root initiation, including pericycle cells and the lateral root meristem (S6C–S6F Fig).

Since (a) mutation of *SCARN* blocked most infections at the epidermis, and (b) *SCARN* was primarily expressed in epidermal cells of infected roots and in the epidermis of young nodules, we tested if epidermal-specific expression could rescue nodule infection in the *scarn-1* mutant. We made a construct in which the epidermal-specific promoter *pEpi* [45] is upstream of the *SCARN* genomic DNA lacking its own promoter. We then generated *A*. *rhizogenes*-transformed hairy roots in the *scarn-1* mutant using this construct (*pEpi*-*SCARN)*. The observed complementation (Fig 1C and Table 2) revealed that *pEpi*-*SCARN* can rescue the infection deficiency, confirming that expression of *SCARN* behind an epidermal-specific promoter is sufficient to permit rhizobial infection.

### *SCARN* expression is regulated by the NIN transcription factor

Mutation of the *NODULE PECTATE LYASE (NPL)* gene induced a block in nodule infection similar to that seen with the *scarn* mutants. The expression of *NPL* is regulated by the *NIN*-encoded transcription factor that is also required for nodulation and infection [30]. Furthermore, the pattern of *NIN* expression is similar to that described here for *SCARN* [46]. We measured (by quantitative RT-PCR) *SCARN* expression in the *nin-1* mutant, revealing that the increased *SCARN* expression caused by *M*. *loti* requires *NIN* (Fig 5A). The NIN-binding nucleotide sequence has been identified [46,47] and analysis of the DNA sequence upstream of *SCARN* revealed three putative NIN-binding sites: one was 1.9 Kb upstream of the predicted translation start and two overlapping sequences of predicted NIN-binding sites are present about 50 bp upstream of the translation start (Fig 5F). We used an electrophoresis mobility shift assay to test if NIN could bind to these *SCARN* promoter regions. Specific retardation was observed with synthetic oligonucleotides corresponding to each of the three *SCARN* promoter regions when they were incubated with the carboxyl-terminal half of the NIN recombinant protein. This region of NIN contains the RWP-RK domain responsible for DNA binding [21]. Gel shift analyses and competition assays confirmed that NIN bound specifically to these promoter regions (Fig 5G).

We also co-expressed 35S:*GFP-NIN* with the reporter fusions *pSCARN*:GUS or *pNIN*:GUS in *Nicotiana benthamiana* leaf cells and GUS activity was determined histochemically and quantitatively in leaf discs. The results indicate that NIN can induce the *SCARN*, but not the *NIN* promoter (Fig 5H). Taken together, these data demonstrate that NIN directly binds to the *SCARN* promoter to activate its expression.

### *SCARN* encodes an unusual protein with a SCAR homology domain and WA domain

The predicted SCARN protein (1627 amino acids) is much longer than the predicted *A*. *thaliana* SCAR1, SCAR2, SCAR3 and SCAR4 proteins (821, 1399, 1020 and 1170 amino-acids respectively) [48]. As with other plant SCAR family proteins, SCARN has an N-terminal conserved SCAR homology domain (SHD), which may mediate the assembly of the SCAR/WAVE complex. It also has a C-terminal predicted WH2 domain connected to an acidic domain (A) which has the potential to bind to G-actin and activate the Actin-Related Proteins ARP2/3 (Fig 1B and S7A Fig). The full-length SCARN protein has only 30% and 26% identity with AtSCAR2 and AtSCAR4 respectively. (For reference, the *L*. *japonicus* and Arabidopsis NAP1 proteins are 77% identical and the PIR proteins are 83% identical; [31]). The SCARN N-terminal SHD and C-terminal WA domains share 67% and 69% identity with AtSCAR2, and 91% and 86% identity respectively with its putative homologue in *M*. *truncatula*. As with the other plant SCAR proteins, SCARN lacks the poly-proline region (PPR) that promotes binding to G-actin binding protein and is normally found in non-plant WASP/SCAR/WAVE family members. Within most plant SCAR proteins there are plant-specific conserved motifs referred to as SCAR of plants central region (SPC) [3,49]. SCARN contains conserved SPC1 and SPC4 domains, but lacks the SPC2 and SPC3 conserved domains (Fig 1B and S7B Fig).

**Fig 6:**
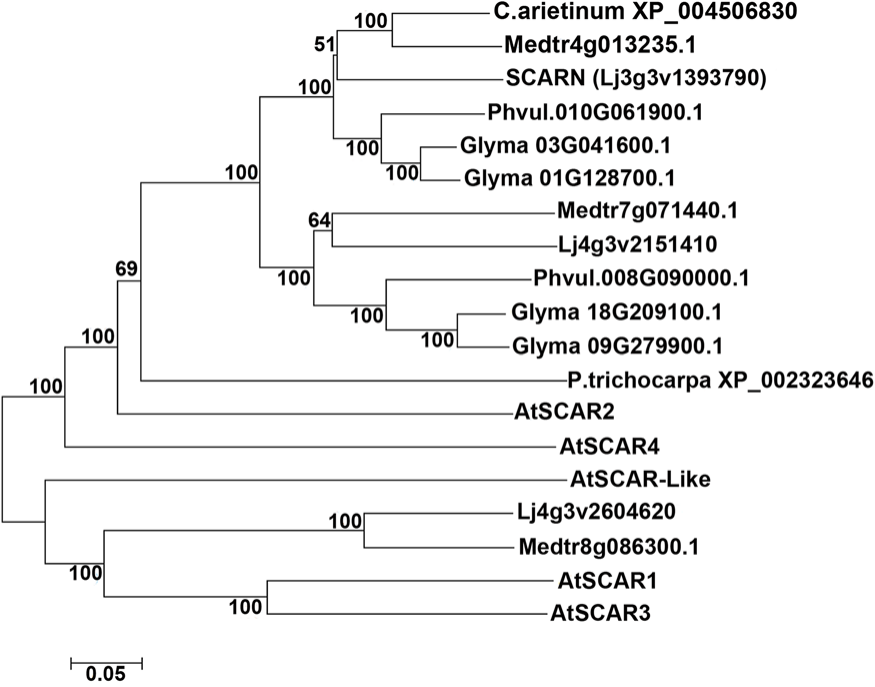
SCARN belongs to a subclade of legume WAVE/SCAR family proteins. The tree shows a phylogenetic analysis of SCARN and other legume SCAR family proteins integrated with SCAR proteins from *Arabidopsis thaliana*. The protein sequence were obtained from TAIR and Phytozome 10.1, and the sequence alignment using ClustalW2, the phylogenetic tree were built using MEGA6.0, by using the neighbor-joining method with the bootstrapping value set at 1000 replications.

We searched for proteins containing conserved SCAR domains in *L*. *japonicus* and other legumes; this identified only three proteins, two of which fell into the same legume-specific clade of proteins. This clade could be split into two subclades, one containing SCARN and another containing a protein clearly related to SCARN. In each sub-clade there is one representative from *L*. *japonicus*, *M*. *truncatula* and *Phaseolus vulgaris* and (as would be expected due to its tetraploid nature) two representatives from *Glycine max* (Fig 6). The SCARN-like protein from *L*. *japonicus* (Lj4g3v2151410) is predicted to be 1461 amino acids long and is 48% identical with SCARN over its full length. The presence in legumes of two subclades of proteins related to SCARN fits with the suggestion [50] that ancestral polyploidy events can lead to enhanced root nodule symbiosis in the *Papilionoideae*. Possibly a gene duplication event may have allowed evolution of SCARN with a specialized role in legume infection.

### The SCARN WA domain binds to ARPC3 *in vitro*

**Fig 7:**
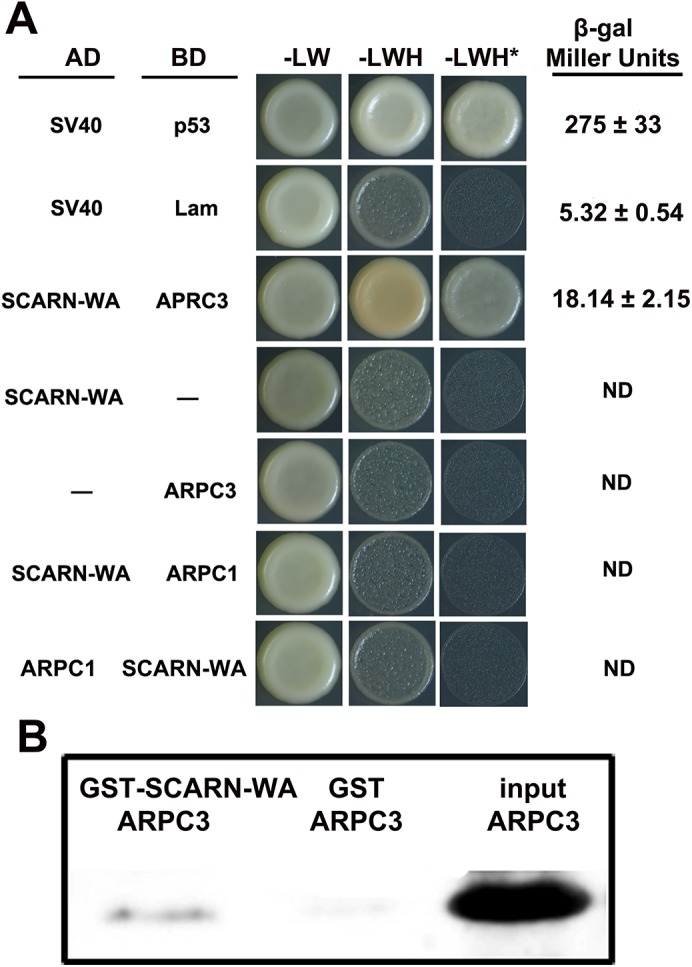
Assays of interaction between the WA domain of SCARN and ARPC1 and ARPC3. **(A)** Yeast-two-hybrid assay of interactions. The SCARN-WA domain was cloned in frame with the yeast Gal4 activation domain (AD). ARPC1 or ARPC3 were cloned in frame with the Gal4 binding domain (BD) and *S*. *cerevisiae* AH109 was co-transformed with the AD and BD plasmids and also transformed with each construct separately. As a positive control, SV40 fused to the activation domain was co-transformed with p53 fused to the binding domain. SV40 and Lam were negative controls. Potential interactions were assayed by comparing growth in the presence (-LW) or absence of histidine (-LWH) and in the absence of histidine in medium containing 10 mM 3-amino-1,2,4-triazole (+ 10 mM 3-AT, -LHW^*^). Quantification of β-galactosidase assays is shown. ND, not determined. **(B)** Pull-down experiments to test interactions *in vitro*. The WA domain of SCARN fused to glutathione transferase (GST-SCARN-WA) or the glutathione transferase (GST) were purified separately from *E*. *coli* and incubated with His-tagged ARPC3 also purified from *E*. *coli*. The GST-SCARN-WA and GST proteins were adsorbed onto glutathione sepharose beads, washed extensively and eluted. The eluents along with the input His-tagged ARPC3 were separated by SDS-PAGE and transferred to a membrane which was stained for His-tagged ARPC3 using antiserum to poly histidine.

The ARP2/3 complex nucleates branched actin filament networks, but requires nucleation-promoting factors to stimulate this activity. In plants, ARP2/3 activation relies on the SCAR/WAVE family and in Arabidopsis the C-terminal WA domain of SCAR2 interacts with the ARP2/3 complex, activating actin nucleation. We tested if the WA domain of SCARN could associate with components of the ARP2/3 complex using a yeast-two-hybrid assay. Since the *LjARPC1* gene is required for legume infection [33], we first tested if the WA domain of SCARN could interact with LjARPC1 in a yeast-two-hybrid assay. Co-expression of the SCARN WA domain fused to the GAL4 binding domain and LjARPC1 fused to the Gal4 activation domain did not permit growth in the absence of histidine. The reciprocal fusion constructs also did not permit growth in the absence of histidine (Fig 7A). These results indicate there is no interaction between the SCARN WA domain and LjARPC1 in the yeast-two-hybrid assay. However, when the GAL4 activation domain fused to the WA domain of SCARN was expressed together with the GAL4 binding domain fused to LjARPC3 (Lj4g3v1934510.1) the yeast strain grew well in the absence of histidine, even in the presence of 10 mM 3-amino-1,2,4-triazole. This indicates LjARPC3 binds to the WA domain of SCARN. Quantitative analysis of the expression of the *lacZ* gene under the control of the *GAL4* promoter confirmed that co-expression of the SCARN WA-GAL4 binding domain and LjARPC1-Gal4 activation domain increased β-galactosidase (LacZ) activity from a background of 5.3 ± 0.5 to 18.1 ± 2.1 units confirming the interaction.

A direct interaction between the SCARN-WA domain and APRC3 was confirmed by *in vitro* pull-down assays. We expressed in *Escherichia coli* a recombinant glutathione S-transferase (GST) fused to the SCARN-WA domain and purified this GST-SCARN-WA recombinant protein using Glutathione Sepharose ^TM^ 4B beads. His-tagged APRC3 was purified from *E*. *coli*, immobilized on nickel–NTA beads and then incubated with the purified GST-SCARN-WA. After washes, proteins retained on the beads were eluted and resolved by SDS-PAGE. The GST-SCARN-WA fusion protein was observed in the eluate by immunoblotting with anti-GST antibody (Fig 7B). The interaction was specific because LjAPRC3 was not pulled down by the GST protein alone (Fig 7B). These results show that the SCARN-WA can interact with ARPC3, suggesting a role for SCARN in the activation of the ARP2/3 complex.

### Early actin rearrangements induced by *M*. *loti* occurs in the *scarn* mutant

Both rhizobial inoculation and addition of purified Nod-factors rapidly induced accumulation of fine bundles of actin filaments in the apical/subapical region of the responding root hairs [9,12,51] and mutation of *L*. *japonicus NAP* and *PIR* genes blocked this effect [31]. Alexa-phalloidin staining of WT and the *scarn-1* root hairs showed similar long cables of actin filaments aligned longitudinally (S8A and S8B Fig), suggesting normal actin arrangements in the *scarn-1* mutant. *M*. *loti* induced an accumulation of actin bundles in region II root-hair tips within 30 min of inoculation of both WT and *scarn-1* mutant; we did not observe any difference in this actin rearrangement in mutant and wild type. We also checked the *M*. *loti*-induced actin rearrangement in root hairs of the *scarn-4* and *scarn-5* mutants using the same technique. About 55% root hairs showed normal accumulation of the actin cytoskeleton and this is consistent with wildtype and *scarn-1* root-hair deformation responses (S8C and S8H Fig and S2 Table). We used the *L*. *japonicus pir* mutant as a control and observed that under the same conditions the *pir* mutation blocked the formation of almost all actin fine bundles in all observed root hair tips (S8D and S8I Fig). Since NIN regulates *M*. *loti*-induced *SCARN* expression, we also checked the *M*. *loti*-induced response in the *nin-1* mutant; this revealed that 60% of the root hairs showed actin accumulation in their tips (S8E and S8J Fig and S2 Table). These results suggest that *SCARN* is not required for the early phase of rhizobial-induced actin cytoskeletal rearrangement in root hairs and is consistent with the observed root-hair deformation in the *scarn* mutants.

### Overexpression of the SCARN SHD domain inhibits nodule infection

**Fig 8:**
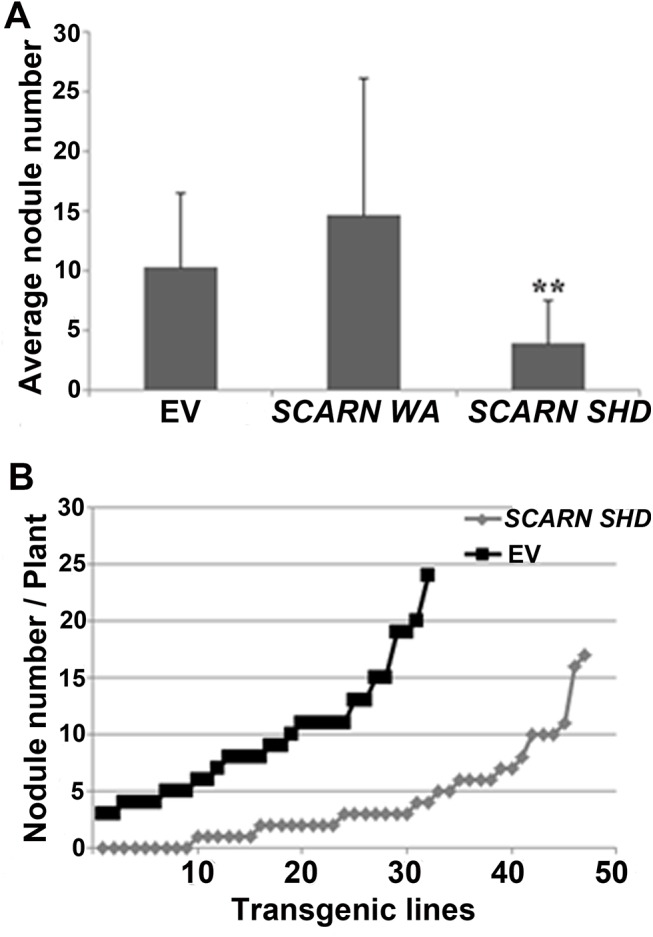
Expression of the Scar Homology Domain (SHD) of SCARN inhibits nodulation. Transgenic hairy roots of wild-type *L*. *japonicus* were generated using *A*. *rhizogenes* carrying transformation constructs for the *in planta* expression of the C-terminal WA (residues 1500–1627) or N-terminal SHD (residues 1–400) of SCARN. The control was roots transformed with the empty vector (EV). Transgenic roots were identified based on the GFP marker on the vector and scored for nodulation 21 d after inoculation with *M*. *loti*. **(A)** Average numbers of nodules (± SD) formed per plant. ** indicates significant difference from the control (p < 0.01) n = 32 (EV), n = 10 (SCARN WA) and n = 47 (SCARN SHD). **(B)** The number of nodules on each of the transgenic root systems of each plant transformed plants with the empty vector (EV) or SCARN SHD domain is shown.

To investigate the importance of the SHD and C-terminal WA domains, we generated constructs that could express either of these SCARN domains using the ubiquitin promoter and introduced them into wild type roots by *A*. *rhizogenes*-mediated hairy root transformation. The constructs encoding the full length SCARN or the C-terminal WH2 domain had no observed effect on nodule formation compared with the empty vector control (Fig 8A). However the expression of the N-terminal SHD domain of SCARN reduced nodule formation (Fig 8A), with about one third of the transformed plants producing no nodules or inducing the formation of only one white bump (Fig 8B). Some of the plants transformed with the SHD domain produced a few pink nodules, but on average the numbers of pink nodules were consistently much lower than that seen with the control plants transformed with the vector *Ubi*:mCherry lacking the SCARN SHD domain (Fig 8A). These results show that strong expression of the SHD domain of SCARN can interfere with normal nodulation, although we observed no effect on root-hair growth. Similar dominant-negative effects have been seen with strong expression of the AtSCAR2 SHD domain causing abnormal trichome development [44]. The observation of a dominant negative effect of the SCARN SHD domain on nodulation (a) confirms the importance of this domain for function and (b) adds weight to the observation that SCARN plays an important role in legume nodulation.

## Discussion

The actin cytoskeleton plays an important role in the polar growth of plant cells, particularly polar-growing cells such as root hairs, trichomes and pollen tubes [52]. There are dynamic interactions and cooperation between the actin cytoskeleton and microtubules [53]. Actin rearrangements are initiated via ARP2/3-mediated nucleation of new actin filaments onto the existing actin filaments; for this to occur the ARP2/3 complex must be itself be activated and this occurs by interactions between the ARP2/3 complex and the SCAR components of the SCAR/WAVE complex [54]. In plants this has been best analyzed in *A*. *thaliana* in which the C-terminal WA domain of the AtSCAR proteins bind to the ARP2/3 complex [43]. There are four SCAR components in Arabidopsis [43,48]; searches for WA domains in Arabidopsis did not identify any additional SCAR components or other potential ARP2/3 activation proteins [3]. There appears to be a degree of functional redundancy between different AtSCAR proteins because double and triple mutations increase the severity of phenotypes [48]. Mutations in AtSCAR2 have the most severe phenotype, causing mildly distorted trichomes [43,44].

The sequence of SCARN suggests it is distinct from the four Arabidopsis SCAR proteins, both in terms of its length and its overall sequence. This distinctiveness fits with the observation that one of its main roles in *L*. *japonicus* is to do with initiation and growth of infection threads during legume infection and nodulation by rhizobia. This conclusion is based on the absence of normal infections in the mutants and by the observation that overexpression of the N-terminal SHD domain of SCARN strongly inhibited nodule infection. In Arabidopsis, it is this SHD domain that enables it to interact with the BRK and ABIL1 components of the SCAR/WAVE complex [44,55]. Presumably the expression of this SHD domain in *L*. *japonicus* root-hairs can titre out the normal SCARN-binding site in the SCAR/WAVE complex, thereby reducing binding by the wild-type SCARN protein. In turn this could decrease the coupling of the SCAR/WAVE complex with the ARP2/3 complex, because we have shown that SCARN can bind to ARPC3 via the conserved C-terminal WA domain in SCARN.

There are two SCARN-like sub-clades in legumes, implying an ancient gene duplication in legumes. Ancestral polyploidization has been proposed to have enhanced development of root nodule symbioses in the papilionoideae [50]. Sequence analysis other SCAR/WAVE complex subunits revealed that orthologues of NAP, PIR or HSPC300 show very high sequence similarity [56], whereas the WASP family proteins (WASH, SCAR etc.) appear to have evolved more rapidly [49,56]. Analysis of SCARN-like proteins suggests that a species-specific gene duplication probably occurred to generate SCARN and a related protein.

In terms of the rearrangements of the actin cytoskeleton, the mutations in *SCARN* appear not to affect the early *M*. *loti*-induced response, whereas mutations in *LjNAP* and *LjPIR* cause defects in the *M*. *loti*-induced rearrangements in the actin cytoskeleton in root hair tip [31]. This difference could be due to some genetic redundancy of SCAR proteins. In Arabidopsis there appears to be some cell-type specificity in the function of SCAR components, probably mostly related to the expression of different SCAR genes in different cell types [37]. It appears from mutant phenotypes and analysis of a reporter-GUS fusion that *LjSCARN* is weakly expressed in the root epidermis and in root hairs, and was more strongly expressed in the epidermis of young nodules. This nodule expression was transient such that in mature nodules expression was only observed in vascular bundles. This is rather different from *NAP*, *PIR* and *ARPC1* expression which was unaffected by *M*. *loti* inoculation [31,33].

The relatively strong *LjSCARN* expression in developing nodules suggests that SCARN plays an important role during nodule development and this role could be related to nodule morphogenesis and/or nodule infection. Although the analyses of actin rearrangements in root hairs of the *scarn* mutants may imply a degree of redundancy, it is nevertheless the case that the *Ljscarn* mutants have a severe infection-thread deficiency. There are two phases of actin nucleation in root hairs [12], one associated with root-hair deformation (mostly changes in the fine actin filaments within 30 min of perception of Nod factors) and one associated with the development of infection threads (within 72 hours after rhizobial inoculation). The lack of infections in the *scarn* mutant may indicate that SCARN plays a significant role in this latter phase of actin rearrangements. It is very difficult to image actin accumulation at this stage in infection-defective mutants, because there are few sites where infections are being initiated and those abnormal infections that do form may be delayed. A few infection threads do initiate in *scarn* mutants, and this is also observed in *nap* and *pir* mutants that are defective for the SCAR/WAVE complex [30]. Understanding how this fits with the late phase of actin rearrangements associated with initiation of infection threads remains to be understood.

One of the unexpected phenotypes of two of the *scarn* mutants was that the root-hairs were branched. The *scarn-1* and *scarn-3* alleles causing this phenotype would both introduce missense translational stops and would be predicted to produce proteins of 1198 and 1099 residues respectively. The observation that the branching was seen in two independent mutants suggests that the phenotype is caused by these alleles. The fact that the phenotype was observed without rhizobial inoculation implies that the SCARN gene must be expressed in root hairs, but the level of expression must be low because we were unable to detect it using the *pSCARN*:GUS fusion. The absence of root-hair branching in the other alleles implies that the phenotype is caused by the presence of the truncated gene product. Intriguingly, branching of root hairs is induced by rhizobial Nod factors and is usually considered to be a consequence of re-initiation of root-hair growth following a pause in growth [8]. The growth of root hairs in some of the mutants appears to be somewhat slower than normal and so it is possible that *scarn-1* and *scarn-3* mutants can periodically show transient inhibition of root hair growth and that pauses in growth stimulate root-hair branching. Why this should be specific to these two but not the other alleles implies an unknown (and unexpected) role for the truncated gene products. Root-hair branching has been associated with action of microtubules rather than actin [57]. Interestingly, in *Dictyostelium*, it was found that the direction of cell migration depended on the action of a microtubule-binding protein to direct SCAR localization, revealing a role for SCAR proteins at the functional interface between actin and microtubules [58]. The root hair branching phenotype observed may therefore indicate a link between SCARN and microtubule function.

The regulation of SCARN probably occurs both during transcription and at the level of activation of the SCAR/WAVE complex. Although the SCARN gene appears to be expressed in epidermal cells prior to the symbiotic interaction, it is induced in response to *M*. *loti*. This is somewhat different from the expression pattern of the *NAP*, *PIR* or *ARPC1*genes which are also required for infection, but appear not to be induced during the symbiosis [31,33]. The enhanced expression of SCARN is regulated by NIN, which is required for the establishment of both nodule development and the formation of the infection foci that precede infection thread growth in root hairs. We checked if other predicted components of the SCAR/WAVE complex are induced during symbiotic interactions and noted that *ABIL1* is induced by Nod factors in *M*. *truncatula* root hairs [7]. This implies that in addition to the normal expression of SCAR/WAVE and ARP2/3 complex proteins, the expression of some of the components is enhanced during early stages of the symbiotic interaction via activation of NIN. NIN is induced by CYCLOPS, which is activated by CCaMK in response to Nod-factor-induced calcium spiking [19]. This NIN-mediated regulation of *SCARN* is consistent with normal mycorrhization in *scarn* mutants, because *NIN* is required for rhizobial but not mycorrhizal symbioses [59].

Post-translational regulation of the ARP2/3 complex occurs in plants and animals [3,60], but in plants the regulation is thought to be less complex in this respect and appears to requires only the SCAR/WAVE nucleation promoting factors and the SCAR/WAVE regulatory complex [3]. One aspect of this regulation in Arabidopsis involves the binding of Rho-related GTP-binding protein ROP2 to PIR1 [61]. ROP-GTPases coordinate vesicular trafficking and cytoskeletal rearrangements during polar growth in Arabidopsis [62]. Nothing is known about the regulation of the ARP2/3 complex in legumes during rhizobial infection, but there is evidence for the action of ROP-GTPases in root-hair growth and rhizobial infection. In *L*. *japonicus* the Nod-factor receptor NFR5 interacts with ROP6, which is involved in infection-thread growth [63]. ROP6 also interacts with clathrin heavy chain1 which is required for normal infection and nodule development, indicating a role for endocytosis during infection and nodulation [64]. In *M*. *truncatula* the Nod-factor receptor NFP interacts with MtROP10 to regulate root hair deformation; overexpression of MtROP10, or a constitutively-active mutant form of MtROP10, leads to depolarized growth of root hairs [65]. More work will be required to establish if these or other ROPs regulate actin nucleation during rhizobial infection and if so, which actin nucleation promoting proteins they bind to.

Although the need for actin rearrangements during rhizobial infection is now well established, the physiological requirements for these are not fully known. One possibility is that the actin cytoskeleton is required for the vesicle trafficking and associated protein targeting required for initiation and maintenance of infection thread growth. If this is correct then the nodulation related pectate lysase NPL would be a likely cargo as would cell-wall and cell-membrane biosynthesis proteins. Another possibility is that actin rearrangements are required for the endocytosis that appears to be required for establishing the symbiosis [64]. Possibly the induced expression of SCARN in both the root epidermis and developing nodule cells points to multiple functions for the actin cytoskeleton during infection and nodule development. It appears likely that one of the plant SCAR proteins has been recruited to take on a specialized role in nucleating actin cytoskeletal changes that play an important role in legume infection and nodule development.

## Materials and Methods

### Biological materials

The *L*. *japonicus* mutants SL2654-3, SL5737-2, SL1058-2 and SL6119-2 were isolated from an EMS mutagenized population of Gifu B-129 [36]. The *scarn-5* allele was obtained from a pool of *L*. *japonicus* mutants with LORE1 transposon insertions [38,39]. *L*. *japonicus* seeds were scarified with sandpaper or immersed for 5–7 min in concentrated H_2_SO_4_ then surface sterilized with 10% NaClO, washed with sterile water and then left to imbibe water. The seeds were then germinated for 4–5 days at 22°C on water agar plates. Seedlings were planted in vermiculite and perlite (1:1) mixed with N-free nutrient (FP) solution [66] and grown under a 16-h/8-h light/dark regime at 23°C. After 5–7 days growth, seedlings were inoculated with *M*. *loti* R7A carrying pXLGD4 (*lacZ*) or pMP2444 (GFP). These strains were grown for 2 days at 28°C in TY liquid containing 5μg/ml tetracycline, at OD_600_≈1.0, pelleted by centrifugation and resuspended in water at OD_600_≈0.01. For hairy root transformation of *L*. *japonicus* roots, *A*. *rhizogenes* strain AR1193 was used, while for *N*. *benthamiana* transient expression, *A*. *tumefaciens* strain EHA105 was used.

### Map-based cloning

Mutants were crossed with MG20, and nodulation phenotype was scored in F_2_ progeny from self-pollinated F_1_ plants. Genomic DNA was isolated and SSR markers were scanned for co-segregation with nodulation defects. Primer sequences and marker information were retrieved from the miyakogusa.jp website (http://www.kazusa.or.jp/lotus/). Rough mapping results indicated that four of the mutations fell within a similar region, so allelism was tested in the crosses described. Fine-mapping was done using SL2654-3 crossed with MG20.

### Analysis of nodulation, infection threads, mycorrhizal infection and actin rearrangement

To score the time course of nodulation, seedlings were grown in N-deficient medium and the numbers of nodules were counted 4 and 5 weeks after inoculation. The number of infection events was determined by microscopy of whole root stained with 5-bromo-4-chloro-3-indolyl-beta-D-galacto-pyranoside (X-Gal), 7 and 14 days after inoculation with *M*. *loti* R7A carrying pXLGD4 (*lacZ*) using at least 15 plants at each time point. For staining, whole roots were immersed in fixative solution (0.1 M potassium phosphate buffer containing 1.25% glutaraldehyde) for 1 h, washed twice for 10 min in 0.1 M potassium phosphate buffer and stained for β-galactosidase activity using staining solution (200 μl 100 mM K_4_Fe(CN)_6_, 200 μl 100 mM K_3_Fe(CN)_6_, 120 μl 2% X-Gal in dimethyl formamide, 3.2 ml 0.1 M phosphate buffer). After staining in the dark overnight at room temperature, roots were rinsed 3 times with water, cleared in 1% NaClO for 1min, then washed 5 times with water. Stained roots were observed using Nikon Eclipse Ni light microscopy under bright-field illumination. Individual infection events were imaged by Nikon digital sight with *lacZ*-marked *M*. *loti*. GFP-marked *M*. *loti-*inoculated roots were counterstained with propidium iodide and analyzed by laser scanning confocal microscopy (Olympus FV1000). Light microscopy of nodule sections was done with nodules fixed in 2.5% (v/v) glutaraldehyde as described [67]. After fixation, tissues were embedded in Technovit 7100 (Kulzer GmbH) resin according to the manufacturer’s instructions and 10 μm transverse sections were taken. Sections were stained with 0.5% (w/v) toluidine blue O in 0.5% (w/v) sodium tetraborate buffer before taking pictures under Nikon Eclipse Ni light microscopy.

For mycorrhizal analysis, the *L*. *japonicus* seedlings were grown in pots with sand and vermiculite (1:1) with sterile *G*. *intraradices* spores at 22°C under a 16h-light /8h-dark cycle. Five weeks after inoculation, roots were treated with 10% KOH for 6 min at 95°C, followed by 3 min in ink at 95°C. Root length colonization was quantified using the grid line intersect method [68] using a Nikon Eclipse Ni light microscopy under bright-field illumination. 15 plants were analyzed for each treatment. Phalloidin staining and microscopy of actin rearrangements was done as described previously [31].

### Root hair deformation

Seedlings prepared and grown as described previously [69] were transferred to slides containing 1ml liquid FP medium and left overnight. The seedlings were inoculated by adding fresh FP medium containing *M*. *loti* R7A (OD600 about 0.01) and then left in the dark for approximately 18 h before analysis. Images were taken using a Nikon DS-Fi2 camera mounted on a Nikon Eclipse Ni light microscope. The branched root hairs observed in the un-inoculated slides were photographed 2 days after transfer of the seedlings to the sterile FP liquid medium slides.

### Complementation, analysis of promoter:GUS expression of SCARN in transformed hairy roots

*SCARN* genomic DNA was amplified from Gifu B-129 leaves using the primer SCARN-attFL-F and SCARN-attFL-R (S3 Table). PCR products were cloned into gateway entry vector pDONR207 and recombined into the destination vector pUB-GW-GFP [40] or *pEpi*-GFP [45]. A 2kb region upstream of the *SCARN* translation start was amplified from Gifu B-129 leaf genomic DNA using primers SCARN-Pro-F and SCARN-Pro-R. The PCR products were cloned into pDONR207 and then recombined into the destination vector pKGWFS7.0 to form *pSCARN*:GUS. The region of *SCARN* encoding the N-terminal SHD domain was amplified by primers SCARN-attFL-F and SCARN-**N-**R; C-terminal WA domain by primer LjSCARN C F and SCARN-attFL-R from Gifu cDNA library. The PCR products were cloned into pDONR207 and then recombined into the destination vector pUB-GW-GFP. All constructs in pDONR207 were confirmed by DNA sequencing, introduced into *A*. *rhizogenes* AR1193 by electroporation and then introduced into roots of wild type or *scarn* mutants by hairy root transformation on half strength B5 medium. The transformed chimeric plants were transplanted into vermiculite/perlite pot and after 5–7 days inoculated with *M*. *loti* R7A containing *lacZ*. The nodulation phenotypes were scored 3 weeks after inoculation after staining for β-glucuronidase or β-galactosidase activity.

### Assays of protein interaction using yeast two hybrid analyses

The SCARN C-terminal WA coding domain, ARPC1 and ARPC3 were all amplified from Gifu cDNA library using the high proofreading enzyme KOD Plus (Toyobo) and the primers LjSCARN-C-F and SCARN-attFL-R, ARPC1-attB-F and ARPC1-attB-R, or ARPC3- attB-F and ARPC3-attB-R respectively. PCR products were cloned into pDONR207, their fidelity was confirmed by DNA sequencing and were then recombined into pDEST-GBKT7 or pDEST-GADT7. The yeast strain AH109 was transformed with the constructs using lithium acetate transformation (Yeast Protocols Handbook PT3024-1, Clontech). Immunoblots were used to validate protein expression using Anti-Gal4 AD (Upstate,Cat. #06–283) and Anti-Gal4 BD (Sigma-Aldrich, 080M4814) antiserum. β-galactosidase activity was assayed following standard methods (Yeast Protocols Handbook PT3024-1, Clontech).

### Pull down of glutathione-fusion proteins

The SCARN C-terminal WA coding domain was amplified from cDNA using the primers SCARN C(SalI)-F and SCARN (Not1)-R and the PCR product was inserted into SalI and NotI digested pGEX4T-1 to form SCARN-WA pGEX4T-1 for expressing a GST-SCARN-WA fusion protein. The *ARPC3* full-length cDNA was amplified using primers ARPC3 (BamH1)-F and ARPC3 (BamH1)-R and the PCR product was cloned into BamHI-digested pET28b, to form ARPC3 pET28b encoding His-tagged APRC3. Plasmids expressing SCARN-WA, ARPC3 and the negative control pGEX4T-1were introduced into *E*.*coli* BL21-Codon Plus (DE3)-RIL (Stratagene). Proteins were induced during exponential growth using 0.5 mM IPTG for 4h at 28°C. The GST-tagged SCARN-WA protein was purified using Glutathione Sepharose ^TM^ 4B beads (GE Healthcare) under native conditions. The purified protein was incubated with soluble His-tagged ARPC3 in 1 mL of interaction buffer (20 mM Tris-HCl, 100 mM NaCl, 0.1 mM EDTA, and 0.2% Triton X-100, pH7.4) for 1 h on ice with gentle shaking and then the mixture was incubated with Glutathione Sepharose ^TM^ 4B beads. The beads were then washed three times with 1.0 mL of NETN100 buffer (20 mM Tris-HCl,100 mM NaCl, 0.1 mM EDTA, and 0.5% NP40, pH 7.4) and three times with 1.0 mL of NETN300 buffer (20 mM Tris-HCl, 300 mM NaCl, 0.1 mM EDTA, and 0.5% NP40, pH 7.4). Retained proteins were eluted following incubation at 100°C for 5 min in 1X SDS sample buffer and analyzed by SDS-PAGE using 12% acrylamide gels. Proteins were transferred from the gel to a PVDF membrane for detection using anti-His antiserum (Abmart).

### RNA extraction and real-time RT-PCR

Total RNA was extracted with TRIpure Isolation Reagent (Aid lab, China) according to the instruction manual and quantified using a Nano-Drop 2000 (Thermo). Reverse transcript first-strand cDNA was synthesized using TransScript one-step gDNA Removal and cDNA synthesis SuperMix (Trans Gen Biotech). Real-time RT-PCR was done with TOYOBO SYBR Green Realtime PCR Master Mix (TOYOBO) and detected using an ABI step-one Plus PCR system. Nodulation marker gene expression samples were generated from whole root of about 10 seedlings of wild type Gifu or *scarn-1* that had been grown for 2 weeks on N-free nutrient solution [66] agar plates and then incubated a further 5 days after inoculation with *M*. *loti* R7A. For analysis of tissue-specific expression, plants were grown in vermiculite-perlite. All of the primers used for qRT-PCR of target transcripts are described in S3 Table quantified relative to the ubiquitin gene as internal control. Data was analyzed as described [30].

### Electrophoresis mobility shift assays (EMSA)

The NIN carrying a C-terminal His tag was purified was described previously [30]. The synthetic biotin-labelled oligonucleotides used for tests of DNA binding are shown in S3 Table. After electrophoresis the gels were developed using the Light Shift chemiluminescent EMSA Kit (Thermo) following the manufacturer’s instructions and the chemiluminescent images were captured using a Tanon-ttoo CCD (Tanon company,China).

### Induction of *pSCARN*:GUS by *NIN* in *N*. *benthamiana*

The full-length *NIN* cDNA was amplified by primers LjNIN-attB-F and LjNIN-attB-R using *L*. *japonicus* cDNA library as template. PCR products were cloned into gateway entry vector pDONR207 and sequenced, then recombined into pK7WGF2 to form LjNIN pK7WGF2. *LjNIN* and *pSCARN*:GUS were introduced into *A*. *tumefaciens* strain EHA105 and infiltrated into *N*. *benthamiana* leaves. Samples were harvested 2 days after agro-infiltration. The GUS activity was measured by GUS staining at least 5 leaves each pair. For fluorimetric GUS assays, about 20 mg frozen leaf tissue was ground by mortar and pestle and protein was extracted using 20 microlitres extraction buffer (50 mM K/NaPO4 buffer pH 7.0, containing 10 mM EDTA (pH 8.0), 10 mM β-mercaptoethanol, 0.1% sarcosyl and 0.1% Triton X-100). Extracts were centrifuged (10000 g, 15 min, 4°C) and the supernatant was used for GUS activity measurement as described with 4-methylumbelliferyl-β-D-glucuronide as substrate (Sigma-Aldrich). GUS activities were measured using DyNA Quantity200 (Hoefer, China). Mean values and standard deviations were determined from three biological replicates.

### Phylogenetic analysis

The *A*. *thaliana* protein sequences were obtained from TAIR (http://www.arabidopsis.org/) and the *L*. *japonicas* protein sequence were obtained from miyakogusa.jp website (http://www.kazusa.or.jp/lotus/) Version 3.0, and other legume SCAR protein sequences were obtained from Phytozome 10.1 (http://phytozome.jgi.doe.gov/pz/portal.html). All the protein sequences are imported into MEGA6.0 [70] for complete alignment using ClustalW2 (http://www.ebi.ac.uk/Tools/msa/clustalw2/). The phylogenetic tree was built using MEGA6.0 [70] and using the neighbor-joining method with the bootstrapping value set at 1000 replications.

## Supporting Information

S1 Fig Positional cloning of a *SCARN* gene. Part of linkage group III of *L*. *japonicus* is shown with markers (TM) and the distances between them are based on the data available at http://www.kazusa.or.jp/lotus/index.html. The clones in the TM0116-TM1465 region are shown as black bars with their names above. The number of recombination events mapped using 439 F_2_ mutant segregants from the cross between *scarn-1* (SL2654-3) and MG20 are shown. Nodulation was scored 3 weeks after inoculation with *M*.*loti* R7A.
(TIF)

S2 Fig Plant and nodule phenotypes of *L*.*japonicus* wildtype and *scarn* mutants. **(A)** Whole plant phenotype of wild-type Gifu B-129 and *scarn-1* five weeks after germination and growth in nitrogen-rich soil. Left: wild-type Gifu B-129; right: SL2564-3 (*scarn-1)*. **(B)** Nodules of wild type Gifu B-129 or *scarn-1*, *scarn-2* and *scarn-3* nodule 3 weeks after inoculation with *M*. *loti* R7A. **(C)** Nodule numbers 2 and 4 weeks after inoculation of wildtype Gifu B-129, *scarn-4* and *scarn-5* plants inoculated with *M*. *loti* R7A: grey columns: mature, pink nodules; white columns: white immature nodule bumps. The 95% confidence intervals are given for each genotype.
(JPG)

S3 Fig The *scarn-1 mutant* is infected by *G*. *irregularis*. **(A)** Wildtype Gifu B-129 or **(B)** *scarn-1*growing in sand were inoculated with *G*. *intraradices*. Five weeks after inoculation the fungus and arbuscules were stained with ink. Bar = 200μm. **(C)** The average numbers of arbuscules wildtype and *scarn-1* were determined using stained sections as in A and B.
(TIFF)

S4 Fig Analysis of expression of several nodulin genes in wild type Gifu and *scarn-1* roots after inoculation with *M*. *loti* R7A. *NIN*, *ENOD40* and *NPL* expression were analyzed in the *scarn-1* mutant roots by quantitative RT-PCR, 5 days after inoculation. The plants were grown on square plates on FP agar medium inoculated (IN) or not inoculated (UN) with *M*. *loti* R7A. Expression levels are shown as means ± SE from three replicates.
(TIF)

S5 Fig The *scarn-1* mutant has normal trichomes, pods and seeds, but slightly shorter root hairs. **(A)** The upper panels show trichomes on fully expanded flower sepals and the lower panels show trichomes on the abaxial leaf midvein. The photographs are of the wildtype Gifu and the *scarn-1*, *scarn-2* and *scarn-5* mutants. Bar = 5mm for all images. **(B)** Scanning electron micrograph of wildtype Gifu and *scarn-1* trichomes formed on the abaxial leaf midvein. Bar = 100 μm. **(C)** Pod and seed of wildtype Gifu and the *scarn-2*, *scarn-4* and *scarn-5* mutants. Bar = 1cm. **(D)** Light microscopy image of uninoculated root segments illustrating the shorter root hairs on *scarn-1 and scarn-5* mutants compared with wildtype Gifu B-129. Bar = 100 micrometers.
(JPG)

S6 Fig *SCARN* expression in different tissues in *M*. *loti* infected and uninfected *L*. *japonicus*. **(A)** Quantitative RT-PCR analysis of the *SCARN* mRNA in leaves, flowers, shoots and roots of *L*. *japonicus*. **(B-F)** Histochemical localization of GUS activity in *A*. *rhizogenes* induced hairy roots transformed with *pSCARN*:GUS. In (B) hairy roots were analyzed 14 days after inoculation with *M*. *loti* R7a/*lacZ* after staining with both Magenta-Gal and X-Gluc to visualize *M*. *loti* (in purple) and *SCARN* expression (in blue). *SCARN* expression was observed in vascular bundles of mature nodule (left) and in whole young nodules (right). (C-F) *pSCARN*:GUS expression is shown in uninoculated plants in (C) a primary root tip, in (D) the lateral root tip, in (E) an initiated lateral root and in (F) in a lateral root elongation regions. Bar = 100 μm.
(JPG)

S7 Fig SCARN has conserved SCAR homology WA domain and two SPC (for SCAR of plants central region) domain. **(A)** SCARN contains conserved SCAR homology domains in N-terminal and WA domain at C-terminal. Amino acid sequences of the SCARN and other WAVE/SCAR family proteins were aligned using ClustalW. The bold black line and the double lines mark two highly conserved blocks toward the N- and C-terminal ends of the SCAR homology domains, respectively. A region enriched in basic amino acids present in all SCAR/WAVE proteins is underlined and labeled. SCARN C-terminal contains the WH2 G-actin binding region and acidic regions are labeled. **(B)** SCARN contains two conserved plant specific motif SPC1 and SPC4.
(JPG)

S8 Fig Alexa-Phalloidin staining of actin filaments in infection zone root hairs of wild type and *scarn* mutants before and after inoculation with *M*. *loti*. Root hairs from the rapidly growing region Ⅱ of root hairs on *L*. *japonicus* seedlings grown on agar plates were imaged following Alexa Phalloidin staining. **(A-E)** show stained root hairs prior to inoculation with *M*. *loti*. **(F-J)** show stained root hairs 30 min after inoculation with *M*. *loti*. (A) and (F) Gifu wild-type root hairs; (B) and (G) *scarn-1*; (C) and (H) *scarn-4*; (D) and (I) *pir;* (E) and (J) *nin-1*. Bars = 10 μm.
(TIF)

S1 Table Allelism test for nodulation. (DOCX)

S2 Table Ratio of *M*.*loti*-induced actin accumulation in the root hair tip. (DOCX)

S3 Table Primer sequences. (DOCX)
