# Supplementary figures and images for: SCARN a Novel Class of SCAR Protein That Is Required for Root-Hair Infection during Legume Nodulation
Source: PLoS Genet. 2015 Oct 30;11(10):e1005623. doi: 10.1371/journal.pgen.1005623 (PMC4627827; doi:10.1371/journal.pgen.1005623)

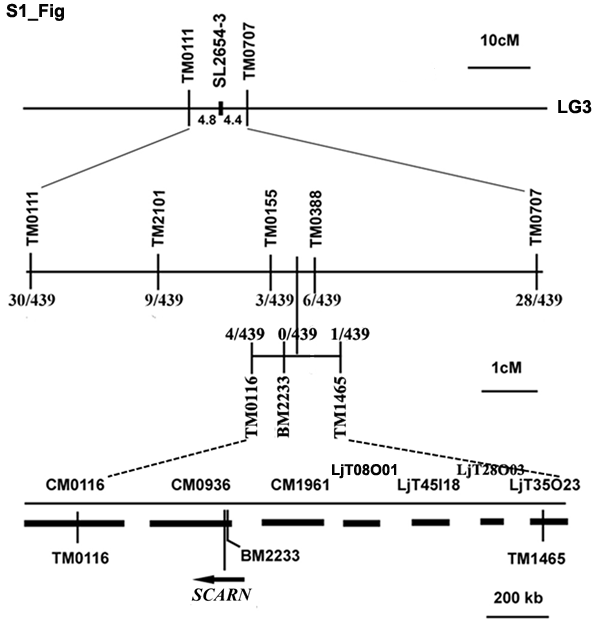

Supplement: S1 Fig — Part of linkage group III of L. japonicus is shown with markers (TM) and the distances between them are based on the data available at http://www.kazusa.or.jp/lotus/index.html. The clones in the TM0116-TM1465 region are shown as black bars with their names above. The number of recombination events mapped using 439 F2 mutant segregants from the cross between scarn-1 (SL2654-3) and MG20 are shown. Nodulation was scored 3 weeks after inoculation with M.loti R7A. (TIF) [file pgen.1005623.s001.tif]

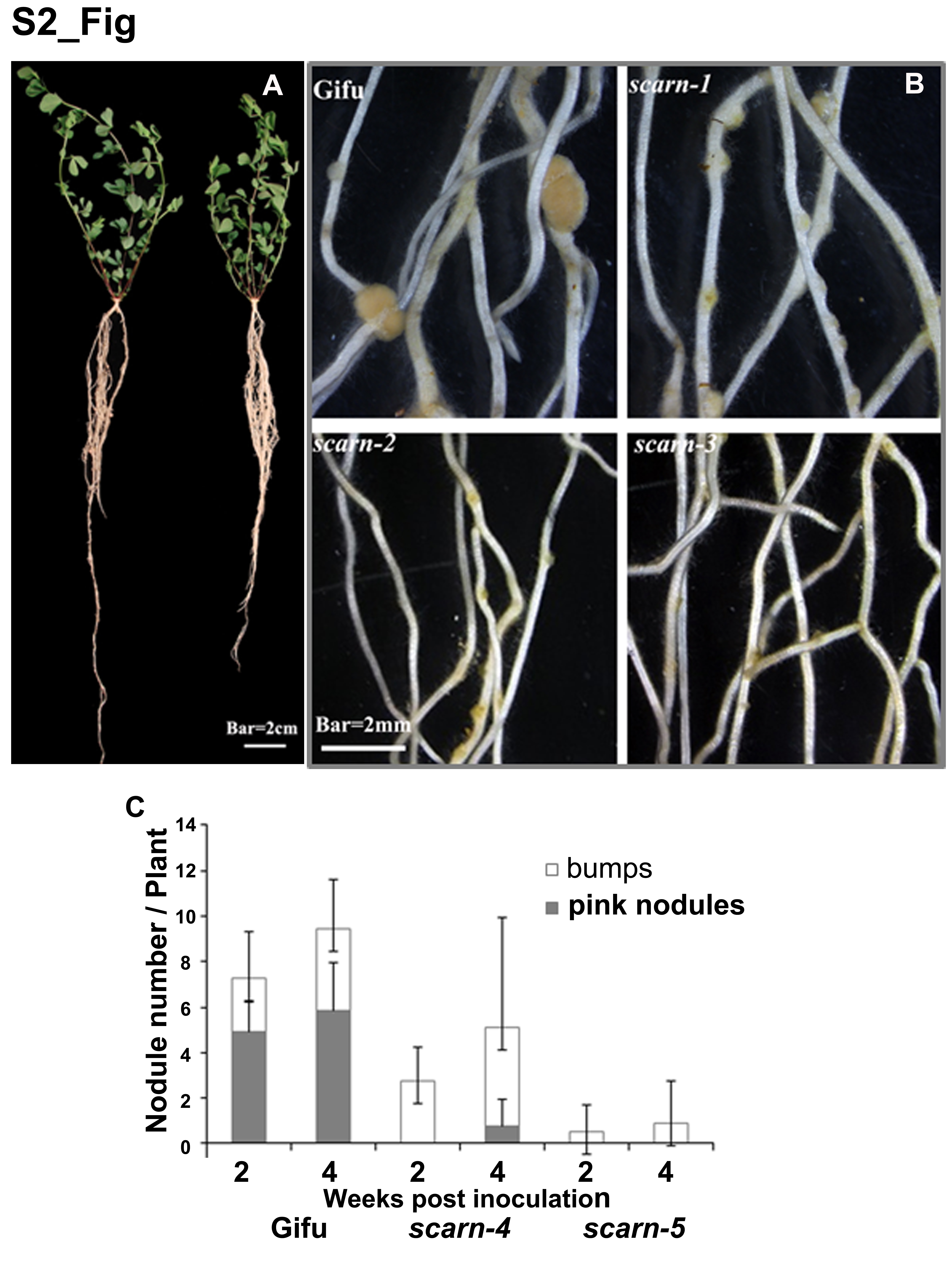

Supplement: S2 Fig — (A) Whole plant phenotype of wild-type Gifu B-129 and scarn-1 five weeks after germination and growth in nitrogen-rich soil. Left: wild-type Gifu B-129; right: SL2564-3 (scarn-1). (B) Nodules of wild type Gifu B-129 or scarn-1, scarn-2 and scarn-3 nodule 3 weeks after inoculation with M. loti R7A. (C) Nodule numbers 2 and 4 weeks after inoculation of wildtype Gifu B-129, scarn-4 and scarn-5 plants inoculated with M. loti R7A: grey columns: mature, pink nodules; white columns: white immature nodule bumps. The 95% confidence intervals are given for each genotype. (JPG) [file pgen.1005623.s002.jpg]

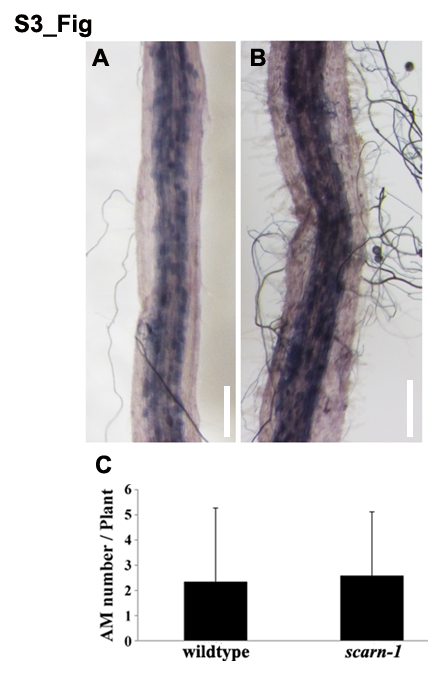

Supplement: S3 Fig — (A) Wildtype Gifu B-129 or (B) scarn-1growing in sand were inoculated with G. intraradices. Five weeks after inoculation the fungus and arbuscules were stained with ink. Bar = 200μm. (C) The average numbers of arbuscules wildtype and scarn-1 were determined using stained sections as in A and B. (TIFF) [file pgen.1005623.s003.tiff]

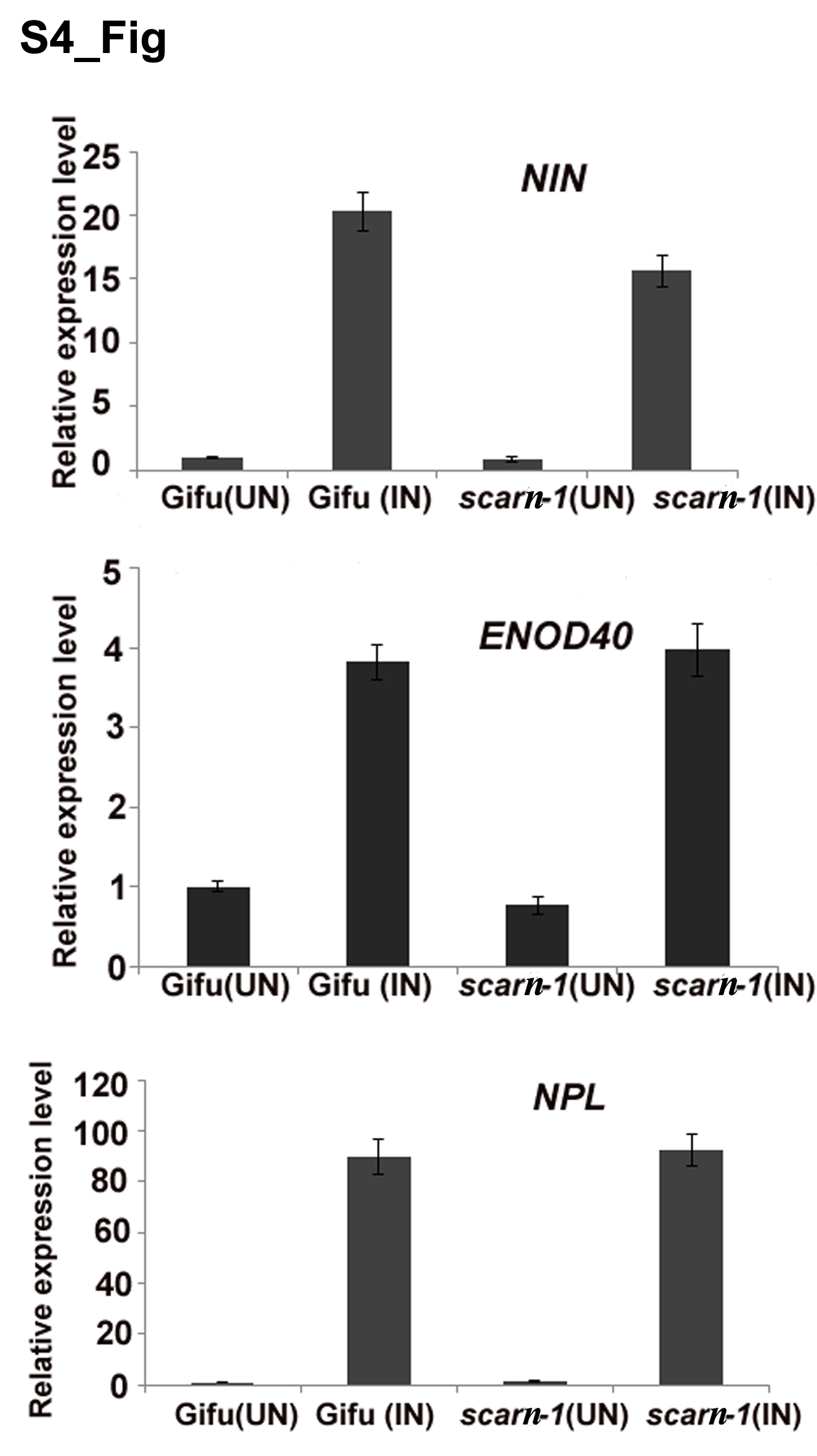

Supplement: S4 Fig — NIN, ENOD40 and NPL expression were analyzed in the scarn-1 mutant roots by quantitative RT-PCR, 5 days after inoculation. The plants were grown on square plates on FP agar medium inoculated (IN) or not inoculated (UN) with M. loti R7A. Expression levels are shown as means ± SE from three replicates. (TIF) [file pgen.1005623.s004.tif]

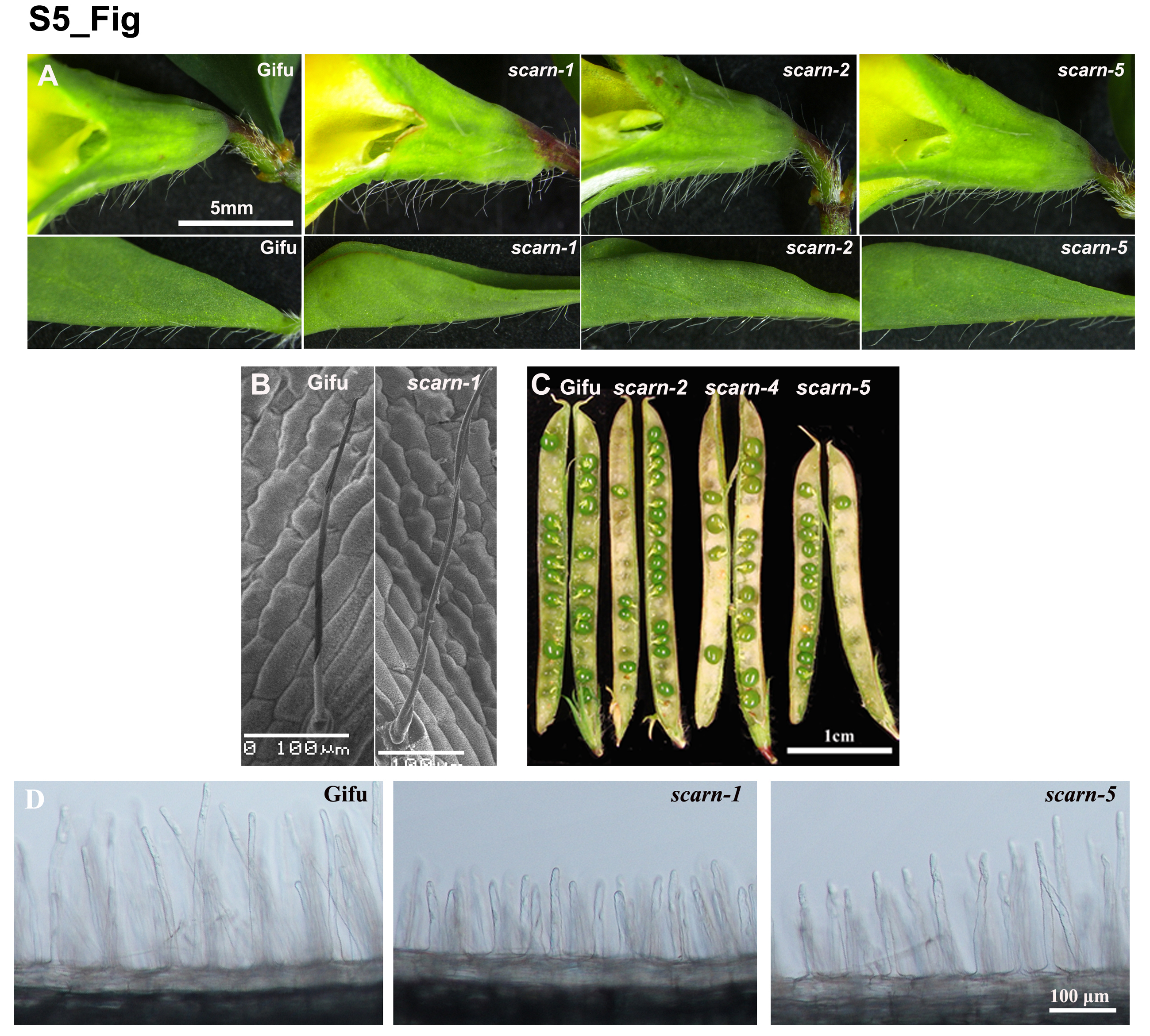

Supplement: S5 Fig — (A) The upper panels show trichomes on fully expanded flower sepals and the lower panels show trichomes on the abaxial leaf midvein. The photographs are of the wildtype Gifu and the scarn-1, scarn-2 and scarn-5 mutants. Bar = 5mm for all images. (B) Scanning electron micrograph of wildtype Gifu and scarn-1 trichomes formed on the abaxial leaf midvein. Bar = 100 μm. (C) Pod and seed of wildtype Gifu and the scarn-2, scarn-4 and scarn-5 mutants. Bar = 1cm. (D) Light microscopy image of uninoculated root segments illustrating the shorter root hairs on scarn-1 and scarn-5 mutants compared with wildtype Gifu B-129. Bar = 100 micrometers. (JPG) [file pgen.1005623.s005.jpg]

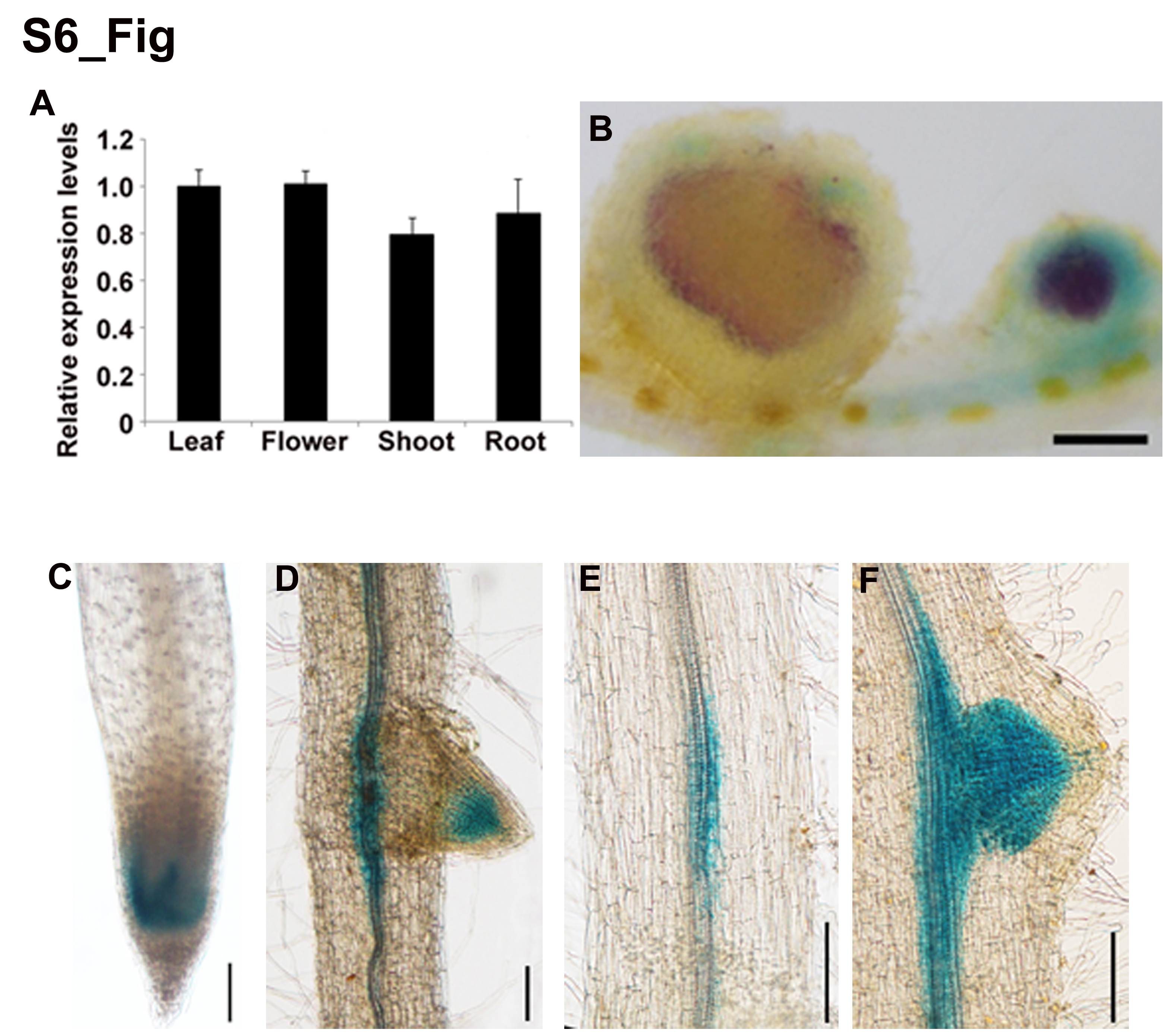

Supplement: S6 Fig — (A) Quantitative RT-PCR analysis of the SCARN mRNA in leaves, flowers, shoots and roots of L. japonicus. (B-F) Histochemical localization of GUS activity in A. rhizogenes induced hairy roots transformed with pSCARN:GUS. In (B) hairy roots were analyzed 14 days after inoculation with M. loti R7a/lacZ after staining with both Magenta-Gal and X-Gluc to visualize M. loti (in purple) and SCARN expression (in blue). SCARN expression was observed in vascular bundles of mature nodule (left) and in whole young nodules (right). (C-F) pSCARN:GUS expression is shown in uninoculated plants in (C) a primary root tip, in (D) the lateral root tip, in (E) an initiated lateral root and in (F) in a lateral root elongation regions. Bar = 100 μm. (JPG) [file pgen.1005623.s006.jpg]

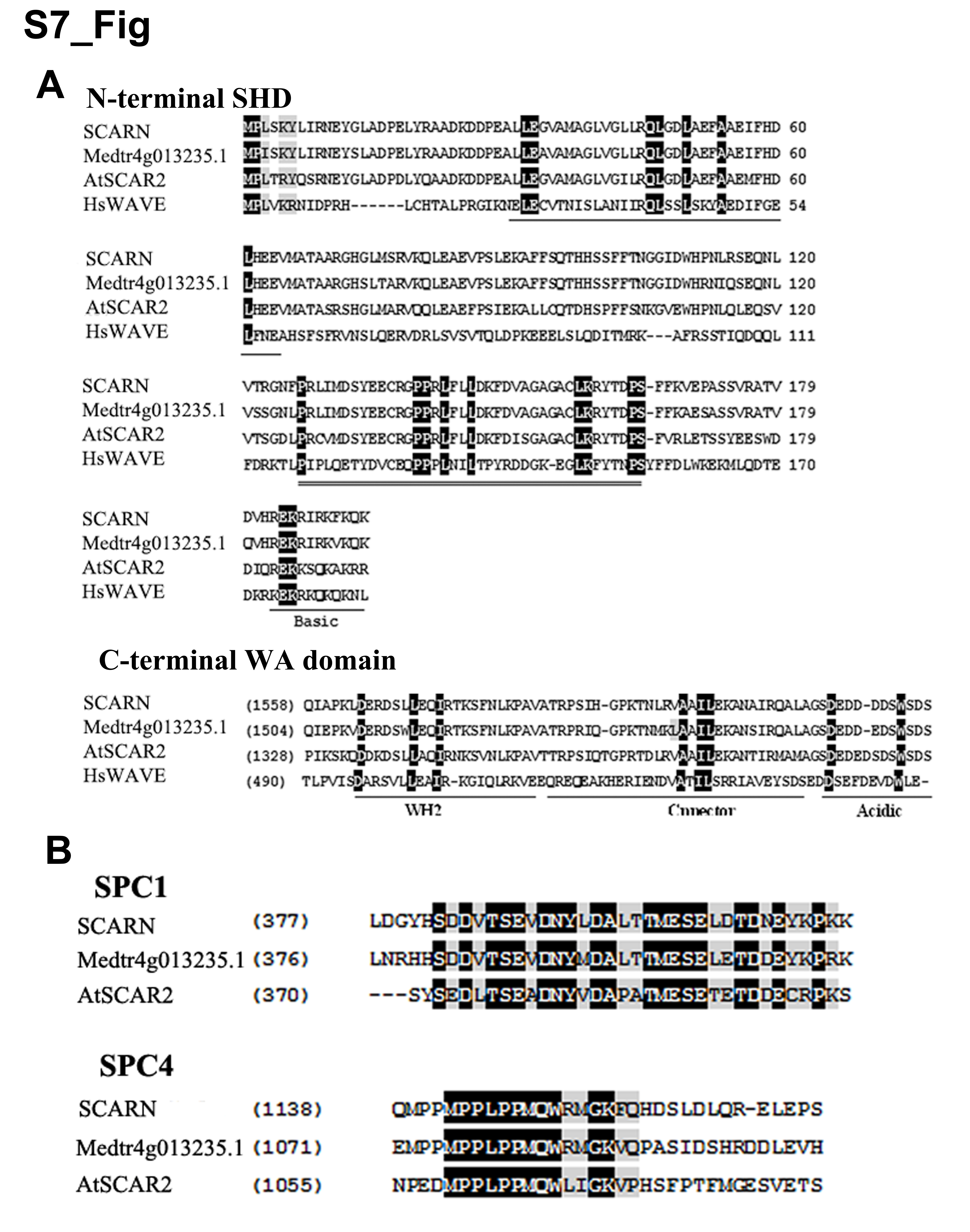

Supplement: S7 Fig — (A) SCARN contains conserved SCAR homology domains in N-terminal and WA domain at C-terminal. Amino acid sequences of the SCARN and other WAVE/SCAR family proteins were aligned using ClustalW. The bold black line and the double lines mark two highly conserved blocks toward the N- and C-terminal ends of the SCAR homology domains, respectively. A region enriched in basic amino acids present in all SCAR/WAVE proteins is underlined and labeled. SCARN C-terminal contains the WH2 G-actin binding region and acidic regions are labeled. (B) SCARN contains two conserved plant specific motif SPC1 and SPC4. (JPG) [file pgen.1005623.s007.jpg]

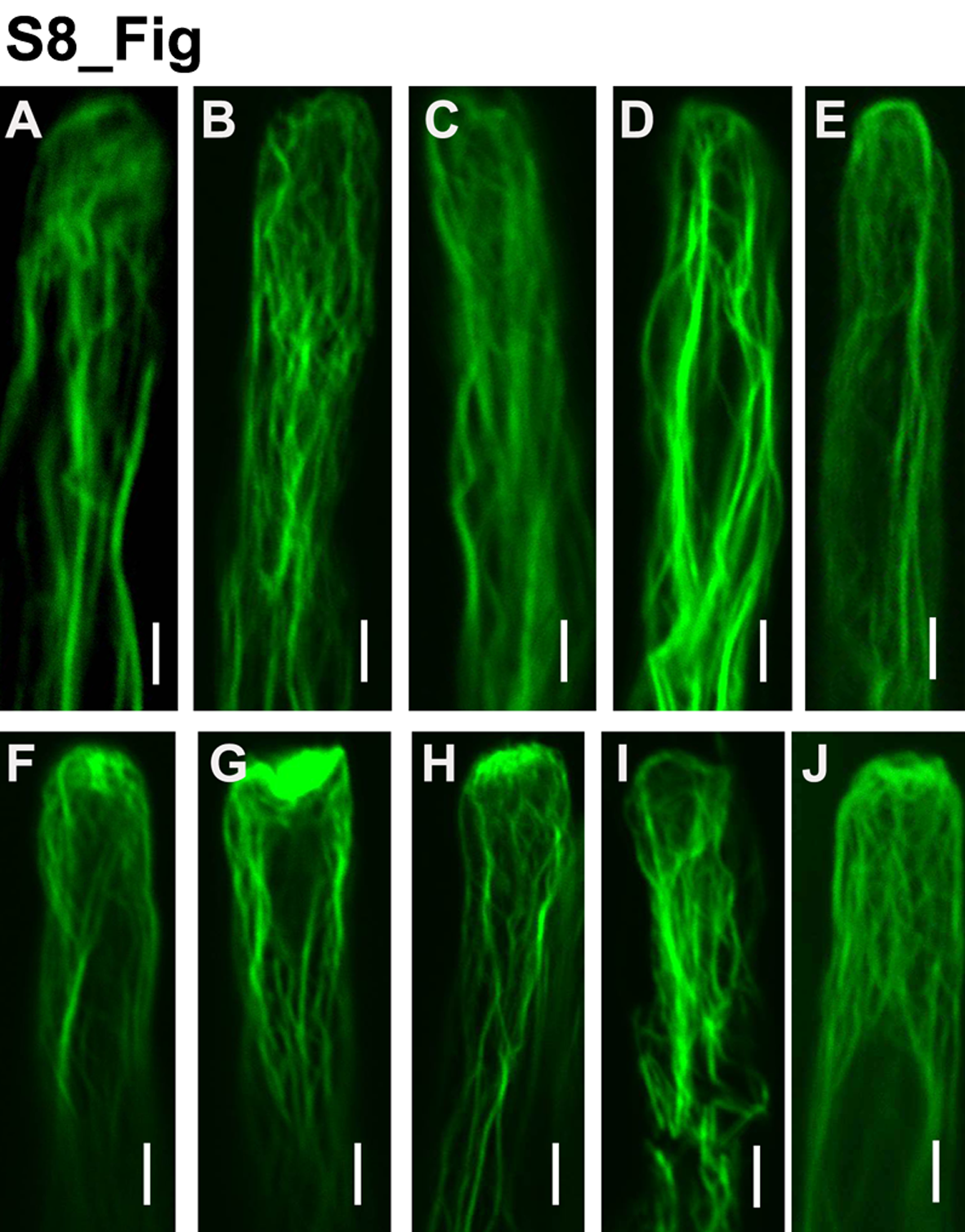

Supplement: S8 Fig — Root hairs from the rapidly growing region Ⅱ of root hairs on L. japonicus seedlings grown on agar plates were imaged following Alexa Phalloidin staining. (A-E) show stained root hairs prior to inoculation with M. loti. (F-J) show stained root hairs 30 min after inoculation with M. loti. (A) and (F) Gifu wild-type root hairs; (B) and (G) scarn-1; (C) and (H) scarn-4; (D) and (I) pir; (E) and (J) nin-1. Bars = 10 μm. (TIF) [file pgen.1005623.s008.tif]
