# Supplementary material for: SCARN a Novel Class of SCAR Protein That Is Required for Root-Hair Infection during Legume Nodulation
Source: PLoS Genet. 2015 Oct 30;11(10):e1005623. doi: 10.1371/journal.pgen.1005623 (PMC4627827; doi:10.1371/journal.pgen.1005623)
Supplement: S1 Table — (DOCX) [file pgen.1005623.s009.docx]

**S1_Table . Allelism test for nodulation**

| Male/Female | SL2654-3 | SL6119-2 |
| --- | --- | --- |
| SL5737-2 | - (6/2) | - (13/3) |
| SL2654-3 | / | - (9/1) |
| SL1058-2 | - (5/1) | / |

- indicates that the F1 plants displayed Nod- or only white bumps phenotype.

6/2 indicate that two independent crosses and have six F_1_ plants
