# Supplementary material for: SCARN a Novel Class of SCAR Protein That Is Required for Root-Hair Infection during Legume Nodulation
Source: PLoS Genet. 2015 Oct 30;11(10):e1005623. doi: 10.1371/journal.pgen.1005623 (PMC4627827; doi:10.1371/journal.pgen.1005623)
Supplement: S2 Table — (DOCX) [file pgen.1005623.s010.docx]

**S2_Table. Ratio of *M.loti*-induced actin accumulation in the root hair tip**

| **Lotus plants** | **Number of form actin-bundle in root hair tips** | **Total number of checked root hairs** | **Ratio** |
| --- | --- | --- | --- |
| Gifu | 69 | 128 | 54% |
| *scarn-1* | 94 | 154 | 61% |
| *scarn-4* | 67 | 122 | 55% |
| *scarn-5* | 87 | 160 | 54.4% |
| *nin-1* | 65 | 117 | 60% |
