# Supplementary material for: SCARN a Novel Class of SCAR Protein That Is Required for Root-Hair Infection during Legume Nodulation
Source: PLoS Genet. 2015 Oct 30;11(10):e1005623. doi: 10.1371/journal.pgen.1005623 (PMC4627827; doi:10.1371/journal.pgen.1005623)
Supplement: S3 Table — (DOCX) [file pgen.1005623.s011.docx]

**S3_Table. Primer sequences**

| Primer name | Use/target | Sequences |  |
| --- | --- | --- | --- |
| SCARN-attFL-F | Transgenic complementation | GGGGACCACTTTGTACAAGAAAGCTGGGTCTCAAGAATCACTCCAACTATC |  |
| SCARN-attFL-R |  | GGGGACAAGTTTGTACAAAAAAGCAGGCTAC ATGCCGCTCTCGAAGTAC |  |
| SCARN-Pro-F | Promoter::GUS | CAAAAAAGCAGGCTatGGCATCAAAGTAGTGTGA |  |
| SCARN-Pro-R |  | GTACAAGAAAGCTGGGTTCTTCTTCTTCGAAATCA |  |
| SCARN-attFL-F | SCARN N-terminal overexpression |  |  |
| SCARN-N-R |  | GTACAAGAAAGCTGGGTCCTACTCCATGGTAGTTAATGC |  |
| SCARN-C-F | SCARN C-terminal overexpression | CAAAAAAGCAGGCTatCTGCCCTTTGAGGGGGAAAT | |
| SCARN-attFL-R |  |  |  |
| SCARN C(SalI)-F | SCARN WA domain protein expression | AGTCGACATATGCTGCCCTTTGAGGGGGAAA |  |
| SCARN (Not1)-R |  | AGCGGCCGCTCAAGAATCACTCCAACTATC |  |
| ARPC3 attB F | ARPC3 Y2H | CAAAAAAGCAGGCTATATGGTTTATCACTCTAGCT |  |
| ARPC3 attB R |  | GTACAAGAAAGCTGGGTCTCAAGGGATAATTATGTT |  |
| ARPC3(BamH1)-F | ARPC3for protein expression | AGGATCCTATGGTTTATCACTCTAGC |  |
| ARPC3(BamH1)-R |  | AGGATCC TCAAGGGATAATTATGTT |  |
| ARPC1 attB1 F | ARPC1 Y2H | CAAAAAAGCAGGCTATATGGGCGGTGATGGCGGTTC |  |
| ARPC1 attB2 R |  | GTACAAGAAAGCTGGGTCTTATAATTCCAATATGTCTTG |  |
| LjNIN-attB-F | LjNIN for *N. benthaminana* transient expression | GGGGACAAGTTTGTACAAAAAAGCAGGCTACATGGAATATGGTTCATTACT |  |
| LjNIN-attB-R |  | GGGGACCACTTTGTACAAGAAAGCTGGGTCTTAAGATGGGCTGCTATTGC |  |
| SCARN qF | Real-time RT-PCR | CATGACAAAAGCAAGCTTAG |  |
| SCARN qR |  | CAGCTAAAGCCTGGCGAATT |  |
| ENOD40 qF |  | AAGAAGCAGATGAGCATTCTCC |  |
| ENOD40 qR |  | TTGGAACAGCACAAGTTGGTAAC |  |
| NIN qF |  | CCAAGCAGCAGTGAATGAGA |  |
| NIN qR |  | AGGAGCCCAAGTGAGTGCTA |  |
| NPL qF |  | CCACATTGCTGGAGGGCCTTG |  |
| NPL qR |  | GCTCACGTACCCACTGCCAC |  |
| UB qF |  | ATGCAGATCTTCGTCAAGACCTTGAC |  |
| UB qR |  | ACCTCCCCTCAGACGAAGGA |  |
| SCARN S1-F | EMSA | GTAGTAGAGAGATAGAGTGAGTGAAAGGGTTTGAGGAATG |  |
| SCARN S1-R |  | CATTCCTCAAACCCTTTCACTCACTCTATCTCTCTACTAC |  |
| SCARN S2-F |  | AAGAGGTGAAACTGAAGGAGTCAAGAGGATTGCAG AAGAA |  |
| SCARN S2-R |  | TTCTTCTGCAATCCTCTTGACTCCTTCAGTTTCACCTCTT |  |
| SCARN S3-F |  | AGTCAAGAGGATTGCAGAAGAAAGTAGGGTTTGTGAATCG |  |
| SCARN S3-R |  | CGATTCACAAACCCTACTTTCTTCTGCAATCCTCTTGACT |  |
